# The Y-Box Binding Protein 1 Suppresses Alzheimer’s Disease Progression in Two Animal Models

**DOI:** 10.1371/journal.pone.0138867

**Published:** 2015-09-22

**Authors:** N. V. Bobkova, D. N. Lyabin, N. I. Medvinskaya, A. N. Samokhin, P. V. Nekrasov, I. V. Nesterova, I. Y. Aleksandrova, O. G. Tatarnikova, A. G. Bobylev, I. M. Vikhlyantsev, M. S. Kukharsky, A. A. Ustyugov, D. N. Polyakov, I. A. Eliseeva, D. A. Kretov, S. G. Guryanov, L. P. Ovchinnikov

**Affiliations:** 1 Institute of Cell Biophysics, RAS, Pushchino, Moscow Region, Russia; 2 Institute of Protein Research, RAS, Pushchino, Moscow Region, Russia; 3 Institute of Theoretical and Experimental Biophysics, RAS, Pushchino, Moscow Region, Russia; 4 Institute of Physiologically Active Compounds, RAS, Chernogolovka, Moscow Region, Russia; University of S. Florida College of Medicine, UNITED STATES

## Abstract

The Y-box binding protein 1 (YB-1) is a member of the family of DNA- and RNA binding proteins. It is involved in a wide variety of DNA/RNA-dependent events including cell proliferation and differentiation, stress response, and malignant cell transformation. Previously, YB-1 was detected in neurons of the neocortex and hippocampus, but its precise role in the brain remains undefined. Here we show that subchronic intranasal injections of recombinant YB-1, as well as its fragment YB-1_1−219_, suppress impairment of spatial memory in olfactory bulbectomized (OBX) mice with Alzheimer’s type degeneration and improve learning in transgenic 5XFAD mice used as a model of cerebral amyloidosis. YB-1-treated OBX and 5XFAD mice showed a decreased level of brain β-amyloid. In OBX animals, an improved morphological state of neurons was revealed in the neocortex and hippocampus; in 5XFAD mice, a delay in amyloid plaque progression was observed. Intranasally administered YB-1 penetrated into the brain and could enter neurons. *In vitro* co-incubation of YB-1 with monomeric β-amyloid (1–42) inhibited formation of β-amyloid fibrils, as confirmed by electron microscopy. This suggests that YB-1 interaction with β-amyloid prevents formation of filaments that are responsible for neurotoxicity and neuronal death. Our data are the first evidence for a potential therapeutic benefit of YB-1 for treatment of Alzheimer’s disease.

## Introduction

Alzheimer’s disease (AD) is a most widespread neurodegenerative pathology. As compared to its hereditary form, sporadic AD occurs nine times more frequently. This could be a result of combination of various factors that eventually lead to similar clinical and morphological pathologies. The major AD features are intra- and extracellular cerebral amyloidosis, intracellular neurofibrillary tangles, a decrease in synaptic density observed in the hippocampus and the cerebral cortex, death of neurons by apoptosis and reactive astrogliosis [1,2]. The severity of cognitive impairments in patients with AD correlates with the extent of the above pathomorphological changes. To date, there are no methods for AD prevention or effective therapy that would allow a considerable delay in AD progression. Therefore, elucidation of the AD molecular mechanisms and revealing new targets for effective AD therapy remain an important issue. To address this problem, valid animal models of AD are required. Several successful genetic models of hereditary AD were produced in transgenic animals that carry mutated genes encoding the brain β-amyloid precursor protein (APP) and presenilins [3]. Adequate models for sporadic forms of AD are very few. The use of olfactory bulbectomy (OBX) for modeling neurodegenerative pathologies similar to sporadic AD is our original concept [4–8]. Presently, this model serves as a research tool for studying neurodegeneration in a number of laboratories [9–12], though previously it was only used as a successful animal model of depression [13]. OBX animals show a number of neuropathological hallmarks typical for AD, i.e., impairment of spatial memory, development of a depression-like state, an increased level of β-amyloid, dysfunction of the acetylcholinergic system, neuronal death in the temporal cortex, hippocampus, and serotonin-synthesizing dorsal raphe nucleus (NRD) of the brain stem, as well as hyperphosphorylation of the Tau-protein [4,6–8,14,15]. It was shown that in OBX guinea pigs, whose β-amyloid amino acid sequence is identical to that of humans, similar deposition of plaque-like aggregates was observed in the brain cortex, white matter, and hippocampus [6,7].

AD falls in the category of chronic diseases with neurodegenerative events occurring much earlier than their clinical manifestation, which is probably caused by activation of compensatory mechanisms. Hence, revealing of the protective mechanisms is of special importance for the design of novel medications aimed at AD prevention and treatment. It was this approach that yielded our previous finding that the heat shock protein 70 (Hsp70) had a pronounced protective effect in OBX animals [16,17].

An analysis of endogenous agents and identification of those with an ageing-caused critical activity decrease highlighted the multifunctional Y-box binding protein 1 (YB-1 or YBX1) [18]. The full-length 324 a.a. YB-1 (YB-1_1−324_) is a member of the family of DNA- and RNA binding proteins with a highly conserved cold shock domain. It participates in a number of cellular events, including proliferation, differentiation, and stress response (see reviews [19,20]); it is also involved in early embryonic development at the stage of neural tube formation [21–23]. Moreover, it has been shown that YB-1 can be secreted from cells by a non-classical mechanism [24]. There is evidence that YB-1 plays an important role in proliferation activation, maintaining the stem cell status, and differentiation of neuronal progenitors [25]. Under oxidative stress, accumulated YB-1 positively regulates stress resistance of the cell and prevents premature ageing [21,26]. It was also shown that extracellular YB-1 is involved in Notch3 signaling and acts as a ligand for Notch3 regulating gene expression [27] and stimulating cell division [28]. In the adult mammalian brain, including human, YB-1 is predominantly localized to neurons of the cortex and hippocampus, while glial cells mostly contain its homologue YB-3 (dbpA, csdA, MSY3/4 and ZONAB) [29]. In mouse organs, the amount of YB-1 decreases with ageing and disappears completely from all organs of old animals, except their liver [18]. Taking into account that many common neurodegenerative diseases, including AD, imply neuronal death, oxidative stress, and a disturbed Notch signaling pathway [30], it may be suggested that a decrease in the amount of YB-1 contributes to these events. The above properties of YB-1 allow it to be considered potentially useful for prevention and/or treatment of neurodegenerative diseases, including AD. This study is focused on the role of YB-1 and its fragments in prevention of behavioral, morphological and biochemical pathologies typical for AD. Our conclusions are based on experimental data from two different mouse AD models.

## Results

### YB-1 suppresses memory impairment in OBX mice and improves spatial learning in transgenic 5XFAD mice

**Fig 1:**
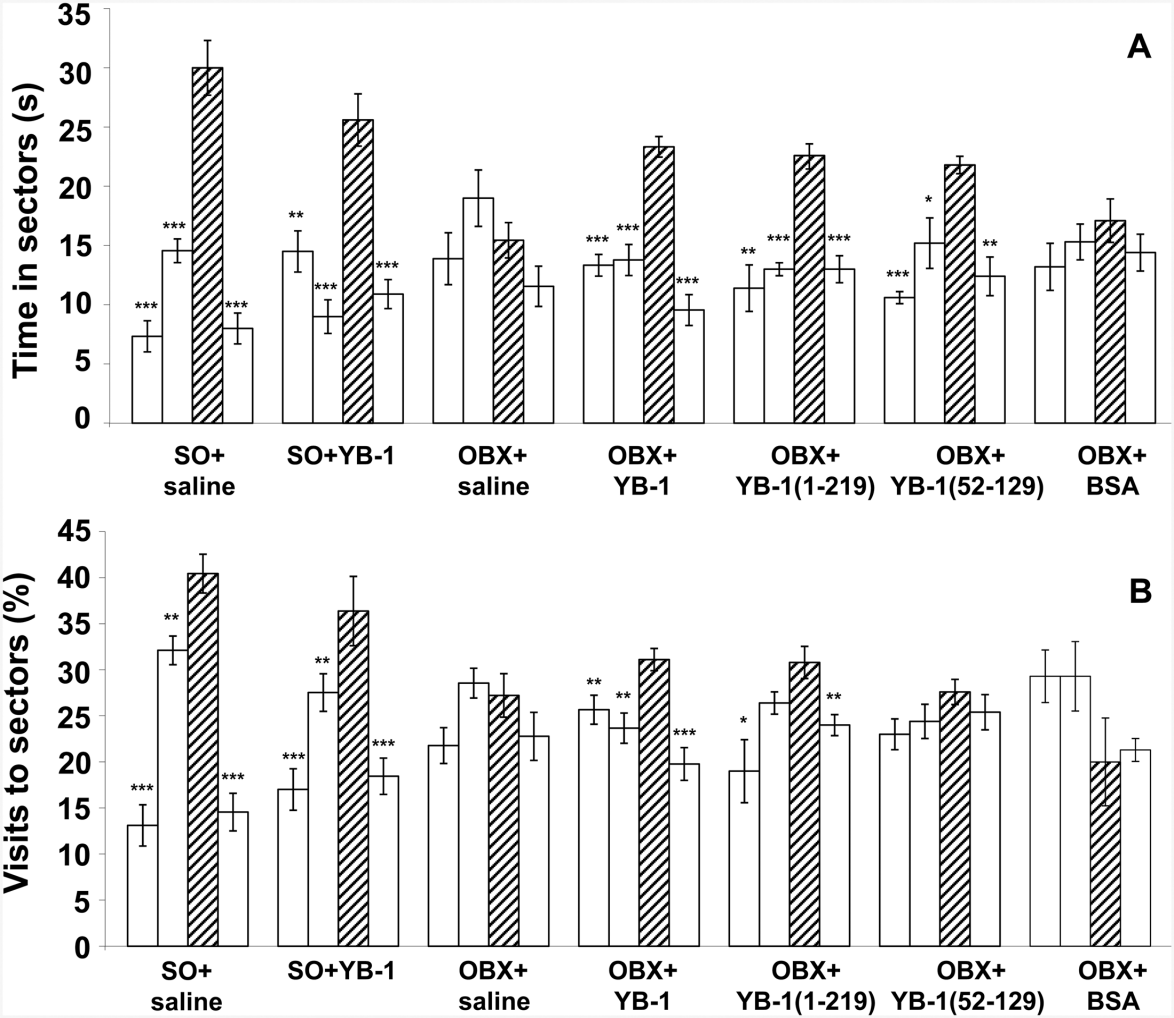
The effect of intranasally injected YB-1 or its fragments on the spatial memory of OBX mice as shown by probe trial tests in the Morris water maze. **A**, Duration (s) of staying in the maze sectors. **B**, Frequency (%) of visiting the maze sectors. Abscissa, categorized groups of experimental animals: SO+saline (n = 9), OBX+saline (n = 9), OBX+YB-1_1-324_ (n = 9), OBX+YB-1_1−219_ (n = 9), OBX+YB-1_52−129_ (n = 9), OBX+BSA (n = 5). Hatched columns represent the target sector. Open columns represent indifferent sectors of the maze. According to the Post hoc analysis with LSD test, significant differences *p <0.05; **p <0.01; ***p <0.001.

First, we elucidated the effect of YB-1 and its two fragments on spatial memory of OBX mice (for one-way ANOVA-based data, see S1 Table; the Post hoc analysis of staying times and visit frequencies for four sectors of the Morris water maze is shown in Fig 1). OBX mice were treated either with full-length YB-1 or one of its fragments (S1 Fig). A considerable impairment of spatial memory was revealed both in YB-1-untreated OBX+saline mice and in those of negative control (OBX+BSA), while OBX mice treated with full-length YB-1 or its fragment YB-1_1−219_ notably preserved their memory and distinguished the sector that during training trials contained the invisible save platform. Importantly, the positive effect was observed for the both memory parameters, i.e., duration of staying in the target (third) sector and frequency of visits to it. For sham operated (SO+saline), OBX+saline, and full-length YB-1-treated OBX mice, time of staying in the target sector was 30.0±2.3>15.4±1.5<23.3±0.9 s, respectively, while respective percentage of visiting it (*vs*. the total number of visits to all sectors) was 40.4±2.1>27.2±2.4<31.1±1.2. The effect of the shortest YB-1 fragment, YB-1_52−129_, was less pronounced, as not all staying times and visit frequencies of the target sector were statistically significantly different from those of indifferent sectors (Fig 1A and 1B).

**Fig 2:**
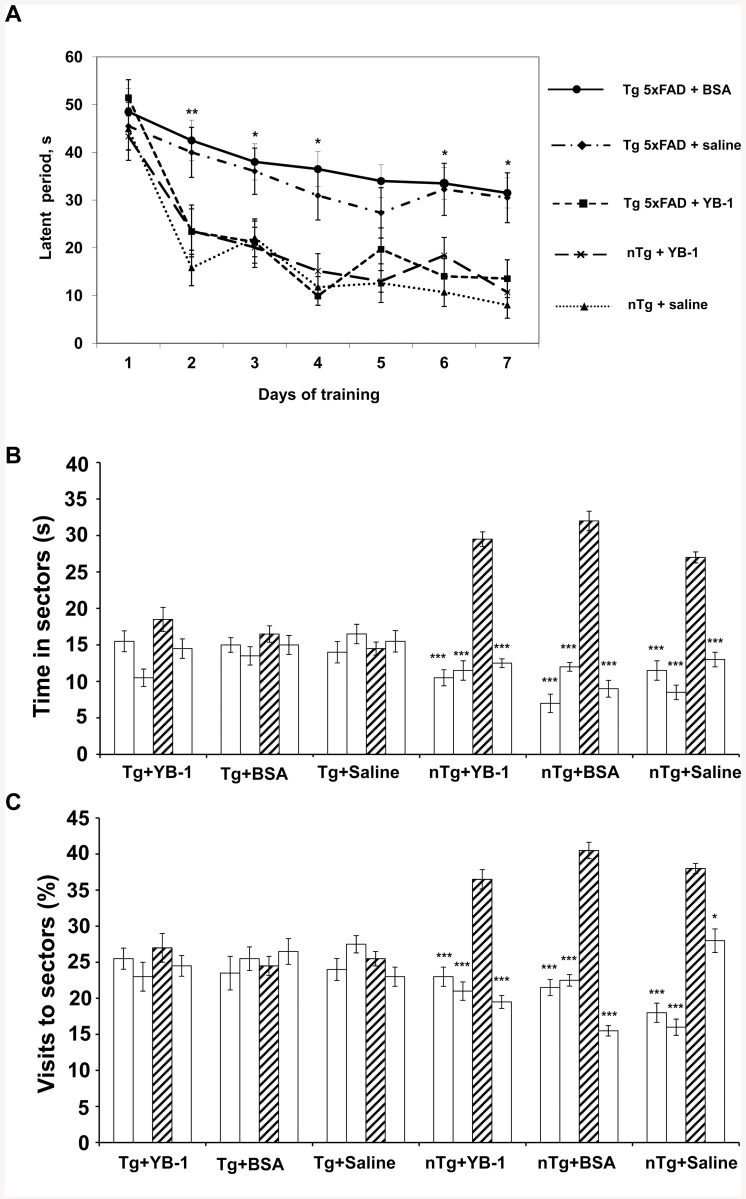
The effect of intranasally injected YB-1 on learning and spatial memory of 6-months-old 5XFAD mice as shown by tests in the Morris water maze. **A**, A positive effect of intranasally injected YB-1 on learning abilities of transgenic 5XFAD mice (Tg+YB-1) as shown by training trials in the Morris water maze. **Abscissa**, the training period (days); **ordinate**, the latent period of finding the invisible save platform (s). **B**, Duration of staying in the maze sectors for the 1 min probe trial period. **C**, Frequency of visiting the maze sectors for the 1 min probe trial period. **Abscissa**, categorized groups of experimental animals. Hatched columns represent the target sector. Open columns represent indifferent sectors of the water maze. According to the Post hoc analysis with LSD test, significant differences *p <0.05; **p <0.01; ***p <0.001.

The most efficient form of YB-1, its full-length form, was further tested on another AD model—transgenic (Tg) 5XFAD mice. The effect of its intranasal administration was studied using 6-months-old transgenic mice with developing AD pathology, i.e., cerebral amyloidosis and plaque formation accompanied by memory loss. A comparative analysis of the results of their training manifested as shortening of the latent period of finding the invisible save platform in the Morris water maze was made for five experimental groups of Tg+saline (n = 5), Tg+YB-1 (n = 6), nTg (n = 5), and nTg+YB-1 (n = 5), and Tg+BSA (n = 6) animals (Fig 2A). YB-1-untreated Tg+saline and Tg+BSA mice demonstrated significantly slower learning abilities, whereas the progress of Tg+YB-1 mice was identical to that of nTg+saline and nTg+YB-1 animals used as controls. However, further memory tests of 6-months-old transgenic mice revealed no positive effect of YB-1 (Fig 2B and 2C). This suggests that only the short-term working memory is positively influenced by YB-1 in Tg mice, but not long-term storage of information.

### YB-1 negatively regulates the level of β-amyloid in the brain of both AD animal models

**Fig 3:**
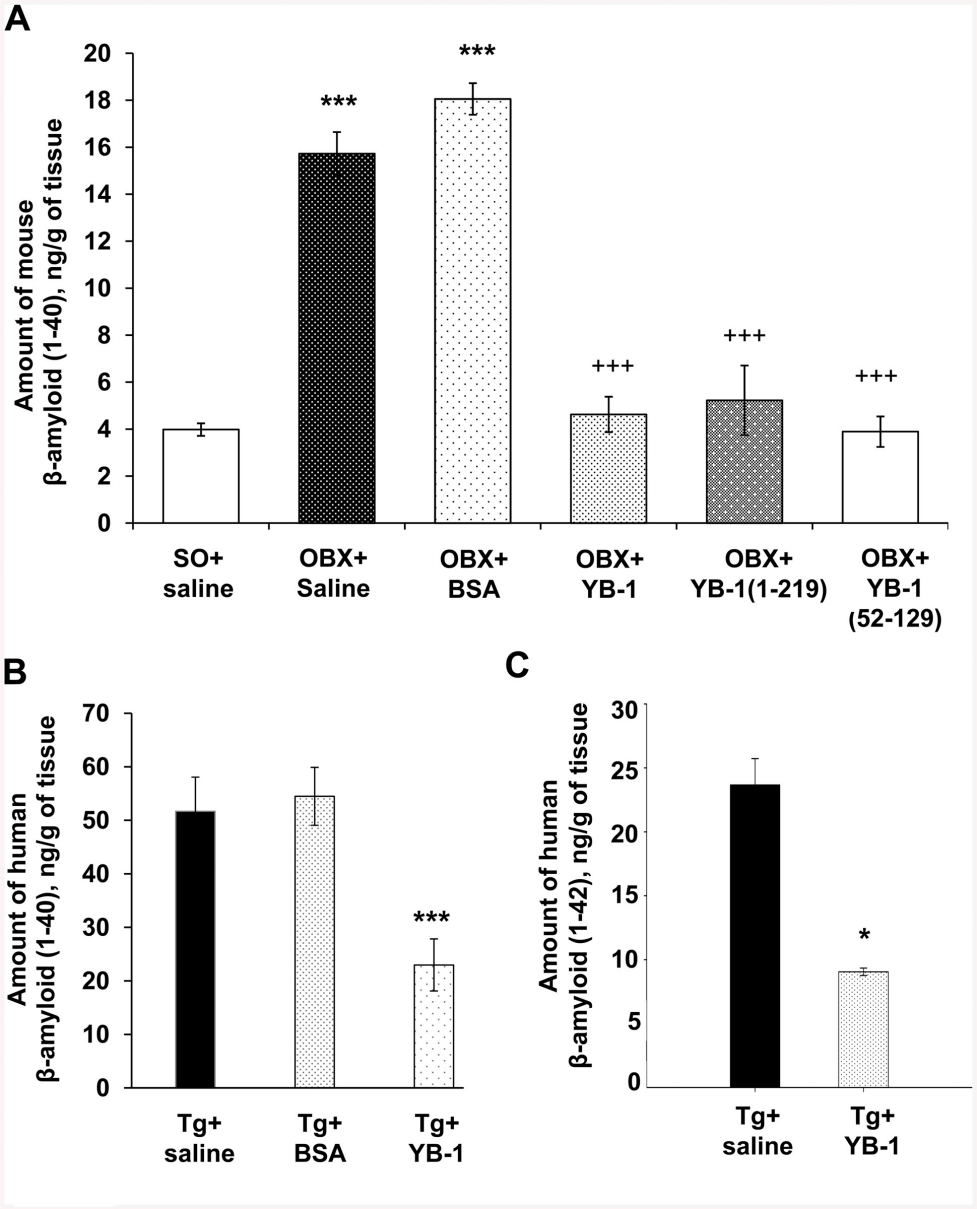
The effect of intranasally injected YB-1 on the amount of β-amyloid in the cortex and hippocampus of OBX mice (A) and 5XFAD mice (B, C). Abscissa, categorized groups of experimental animals. Ordinate, ELISA-estimated amount of mouse β-amyloid (1–40) in (A) and human β-amyloid (1–40) and (1–42) in (B, C) (ng per g of tissue (cortex+hippocampus)). According to the two-tail Student-test, for SO+saline (A) and Tg+saline (B, C) mice, the significant difference *p<0.05, ***p <0.001; for OBX+saline (A), the significant difference ^+++^ p < 0.001; β-amyloid values are given as mean±SEM.

To elucidate the molecular mechanisms of the effects of YB-1 and its fragments on neurodegeneration in OBX mice, we investigated whether and how the β-amyloid level is affected by the presence of YB-1. In a set of *in vitro* experiments we demonstrated that in extracts from the neocortex + hippocampus of YB-1-treated OBX mice the amount of β-amyloid (1–40) remained identical to that of SO mice (4.0±0.3 ng/g), and thus was considerably lower than that of YB-1-untreated OBX+saline and OBX+BSA mice (15.8±0.9 ng/g and 18.05±0.73 ng/g of tissue, respectively; for both p<0.001) (Fig 3A). In 6-months-old 5XFAD mice, three-weeks-long treatment with YB-1 produced a more than 2-fold β-amyloid (1–40) and β-amyloid (1–42) decrease in extracts from the neocortex and hippocampus, in contrast to transgenic animals treated with saline or BSA that showed no such effect (Fig 3B and 3C). Interestingly, the level of β-amyloid (1–40) in transgenic mice observed after intranasal administration of YB-1 was considerably higher than the level of β-amyloid (1–40) in the brain of OBX+YB-1 mice, which probably explains the absence of the positive YB-1 effect on spatial memory in transgenic mice.

### YB-1 suppresses progression of pathology in neurons of OBX mice

**Fig 4:**
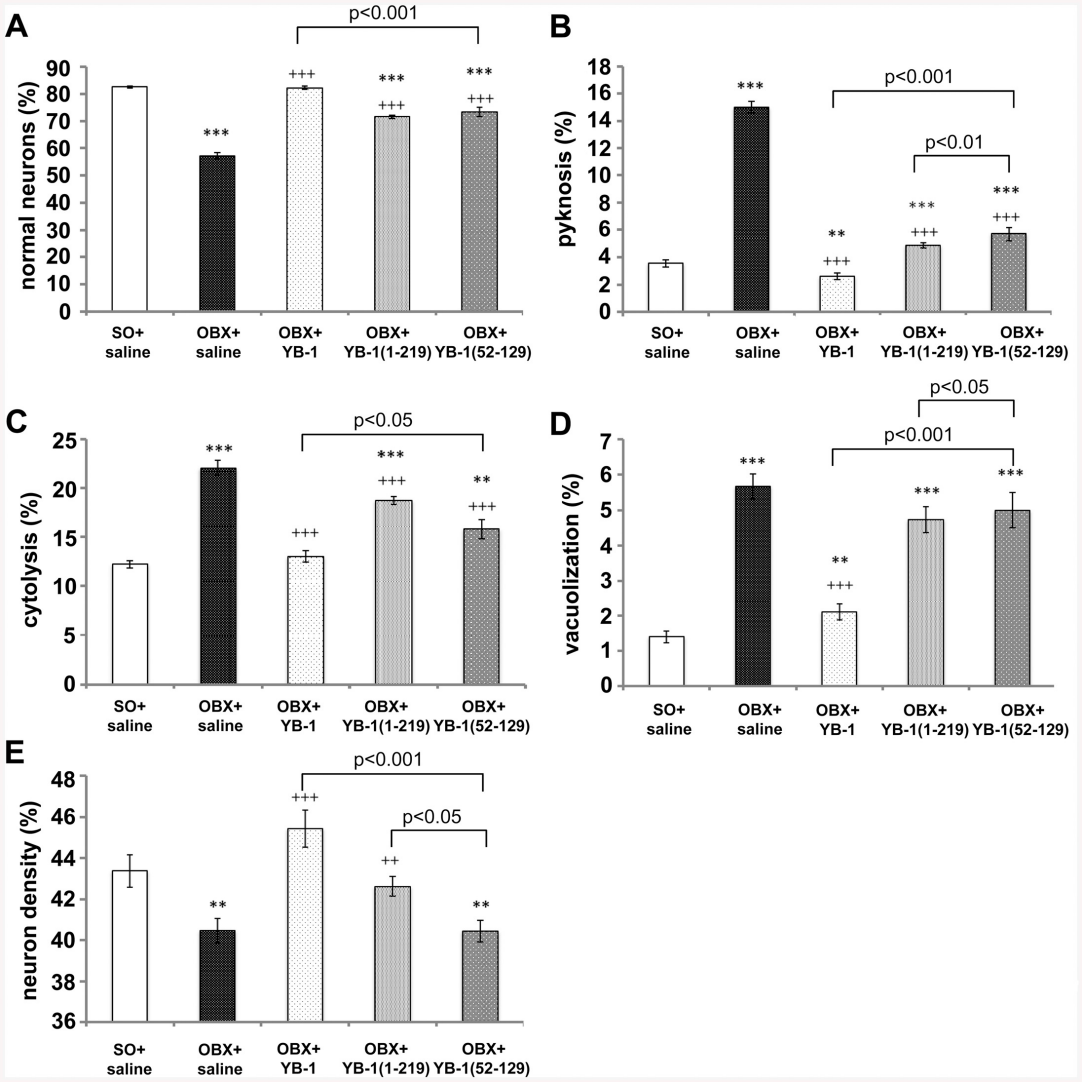
Comparison of the positive effect of YB-1 and its two fragments on the morphofunctional state of neurons in the temporal cortex of OBX mice. **A,B,C,D**, ordinate, percentage of normal cells and those with indicated pathology: pyknosis, cytolysis, and vacuolization, respectively. **E**, ordinate, neuronal density (n/mm^2^). **A,B,C,D,E**, abscissa, categorized groups of experimental animals: SO+saline (n = 5), OBX+saline (n = 5), OBX+YB-1_1-324_ (n = 9), OBX+YB-1_1−219_ (n = 5), OBX+YB-1_52−129_ (n = 5). Significant differences for SO mice are denoted by asterisks (*), while those for YB-1-untreated OBX mice are daggered (^+^): ***p or ^+++^p<0.001; **p or ^++^p<0.01; *p or ^+^p<0.05.

The severity of cognitive impairments in patients with AD correlates with changes in neuronal morphology, and specifically, with the level of neuronal death. Therefore, we studied the effect of YB-1 by assessment of the morphofunctional state of neurons in memory-responsible regions of the brain, namely, in the temporal cortex and hippocampal areas. We analyzed neuronal density and percentage of normal neurons *versus* cells with pathological changes, including pyknosis, cytolysis, and vacuolization (S2 Fig), in SO, OBX, and YB-1-treated OBX mice. The results of these analyses are presented in Table 1. Since in all previous experiments the effect of OBX+BSA did not differ from that in the OBX+saline group, the results shown by OBX+saline mice were used as a control in analyzing the YB-1 effect on the state of neurons of OBX mice. As seen, bulbectomy induced an increase of pathological neurons from 15–18% to 33–43% in all studied brain structures, with a critical growth of the number of pyknomorphic neurons, which reflects the death rate among neuronal population. Also, an increased number of cytolysis-type cells with damaged outer membranes were observed. In OBX mice, administration of full-length YB-1 had a positive effect on the state of neurons that was manifested as a reduced number of pyknomorphic and cytolysis-type cells in the studied brain regions. This enlarged the number of functionally normal neurons in the cortex and hippocampal areas. The highest positive protective effect of YB-1 was observed in the temporal cortex, where neuronal density and percentage of normal neurons were close to those of SO animals. Fig 4A–4E) shows percentage of normal neurons and various pathologies (pyknosis, cytolysis, and vacuolization) observed in the neocortex of OBX mice treated with full-length YB-1 or its fragments, as compared with SO or YB-1-untreated OBX mice. It is clearly seen that although all three agents, YB-1 and its two fragments, suppressed progression of pathological changes in neurons, the most pronounced effect was produced by full-length YB-1.

**Table 1:** The effect of intranasally injected full-length YB-1 on neuron density and the morphofunctional state of neurons in the temporal cortex and hippocampus of OBX mice.

Brain	Group	Neuron density	Normal neurons,	Pathological neurons, %		
region			%	Pyknosis	Cytolysis	Vacuolization
**Temporal cortex**	SO+saline (n = 5)	1204.86±21.94	82.65±0.4	3.58±0.27	12.25±0.38	1.4±0.16
	OBX+saline (n = 5)	1124.07±16.51**	57.27±1.06***	15.03±0.43***	22.03±0.78***	5.67±0.35***
	OBX+YB-1 (n = 9)	1262.04±24.94^+++^	82.27±0.64^+++^	2.6±0.24**^+++^	13.03±0.55^+++^	2.1±0.23**^+++^
**Hip CA1-CA2**	SO+saline (n = 5)	2797.22±24.37	86.65±0.54	2.03±0.23	9.95±0.43	0.95±0.17
	OBX+saline (n = 5)	2603.70±25.69***	67±0.75***	7.73±0.32***	21.37±0.68***	3.93±0.23***
	OBX+YB-1 (n = 9)	2685.19±26.78**^+^	78.03±0.64***^+++^	3.7±0.24***^+++^	15.7±0.60***^+++^	2.53±0.24***^+++^
**Hip CA3-CA4**	SO+saline (n = 5)	2129.86±29.49	84.9±0.6	2.18±0.25	11.5±0.52	1.43±0.17
	OBX+saline (n = 5)	1936.11±20,24***	64.07±0.81***	6.83±0.22***	24.27±0.63***	4.83±0.25***
	OBX+YB-1 (n = 9)	1993,52±15,11***^+^	73,43±0,46***^+++^	5,1±0,32***^+++^	17,23±0,34***^+++^	4,23±0,29***

The two-tail t-test p-values for SO mice are *p < 0.05; **p < 0.01; ***p < 0.001, and those for OBX mice are ^**+**^p < 0.05; ^**++**^p < 0.01; ^**+++**^p <0.001.

### YB-1 decreases the number of amyloid plaques in the brain of transgenic 5XFAD mice

**Fig 5:**
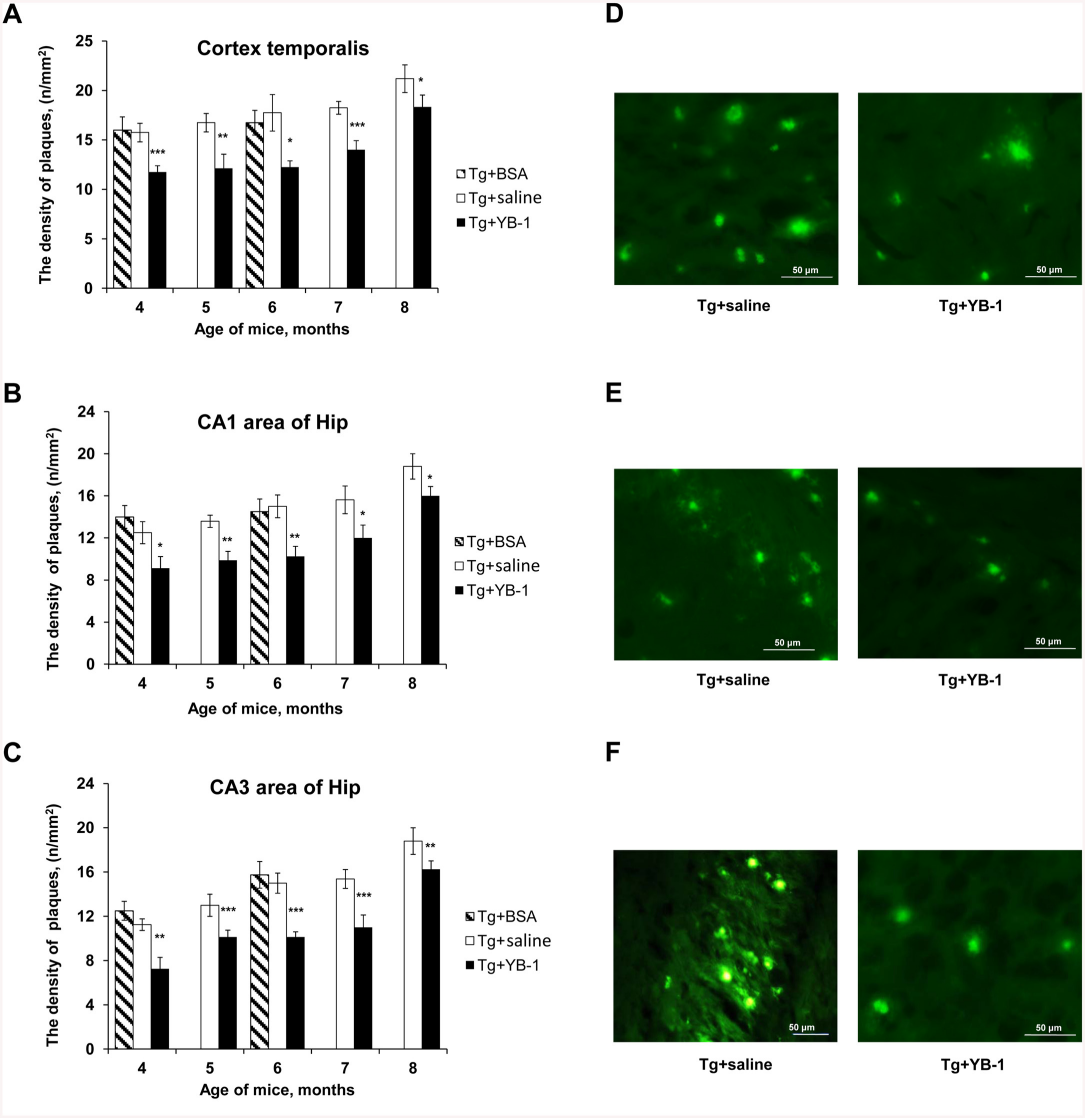
The effect of intranasally-injected YB-1 on temporal dynamics of plaque density in the brain of transgenic 5XFAD mice. **Abscissa**, age of mice (month); **ordinate**, plaque density (n/mm^2^) in view field (x10). Filled columns represent data for 5XFAD mice intranasally administered with YB-1; open columns represent data for control Tg mice injected with saline; hatched columns represent data for Tg+BSA mice. **A,B**, and **C**, Plaque densities in the cortex and hippocampal areas CA1 and CA3, respectively. **D,E**, and **F**, representative microphotographs of the cortex, CA1, and CA3 respectively, for Tg+saline (left column) and Tg+YB-1 (right column) mice. The data are given as m±SEM. Significant differences for Tg+saline mice in each age group are denoted by asterisks (*): ***p<0.001; **p<0.01; *p<0.05.

Staining of β-amyloid-containing plaques with Thioflavin S was used to monitor the amyloidosis dynamics in transgenic 5XFAD mice of various age and the possible effect of YB-1. YB-1 was daily injected intranasally to mice of all age groups during a period of three weeks. Matching age control groups received saline, and two groups of transgenic mice (3- and 5-months-old) were treated with BSA and used as an additional negative control. Fig 5A, 5B and 5C presents a quantitative analysis of amyloid plaque density in the cortex and two hippocampal areas CA1 and CA3 of 5XFAD mice from different age groups. It is seen that, as compared to controls, all YB-1-treated mice exhibited a reduced number of plaques in all studied brain regions and at all ages. Fig 5D, 5E and 5F demonstrates a decrease in the plaque number, as well as blurriness and muted glow of the remaining plaques in the brain of 5XFAD mice after YB-1 treatment.

**Fig 6:**
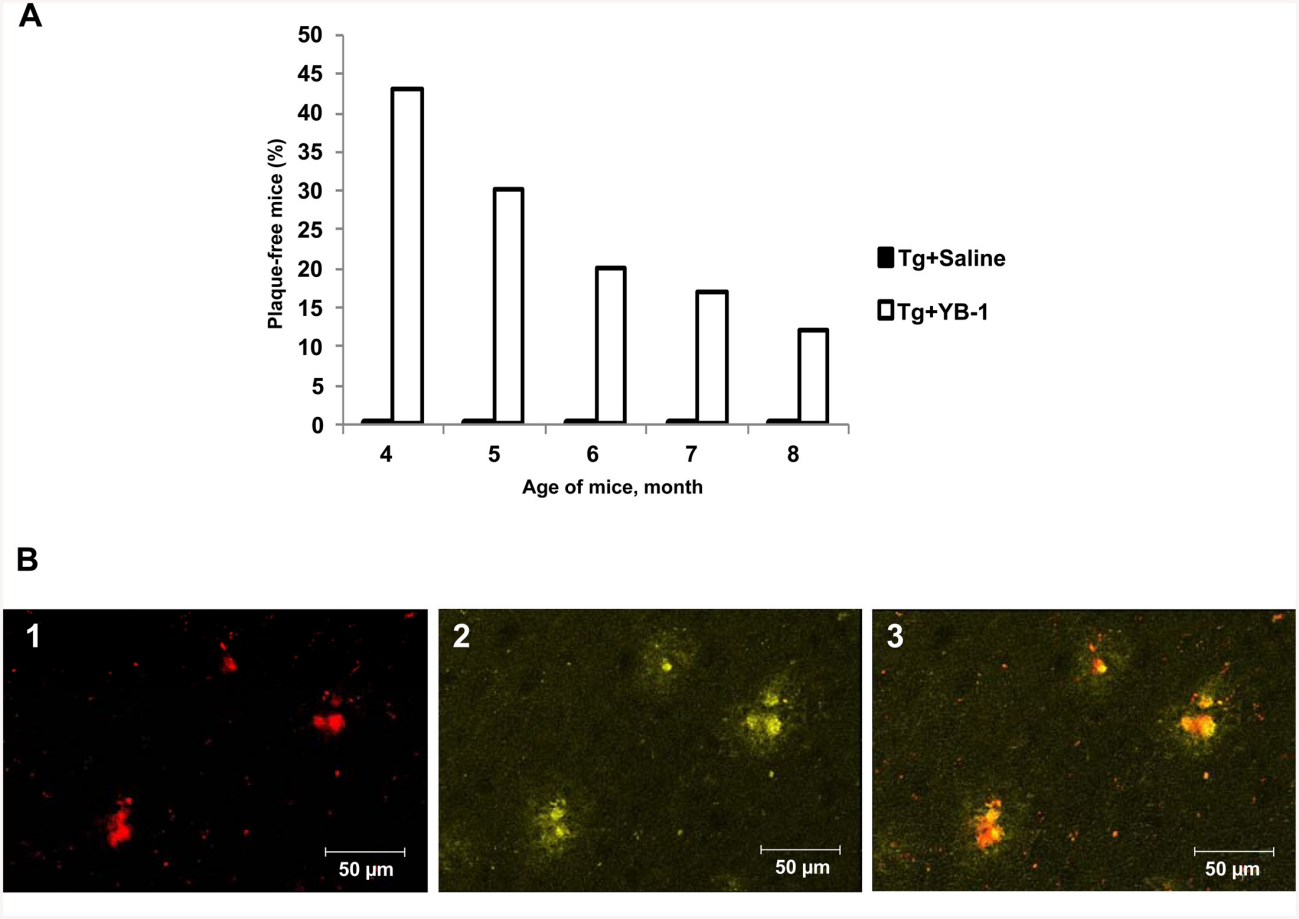
Plague-free 5XFAD mice and the amyloid nature of plaques in transgenic mice. **A**, Age-dependent decrease of percentage of plaque-free transgenic 5XFAD mice treated with YB-1. Abscissa, the age of mice; ordinata, percentage of plaque-free mice. **B**, Plaques in the brain of Tg mice contain β-amyloid. In brain slides, β-amyloid was stained with monoclonal Anti-Human β Amyloid Protein antibody (**1**), plaques were stained with Thioflavin S (**2**) as described under Materials and Methods. (**3**), merged staining.

Interestingly, in some transgenic mice with intranasally injected YB-1 no amyloid plaques at all were observed in any studied brain region. We emphasize that each control transgenic mouse (Tg+saline) or (Tg+BSA) had plaques in these brain regions at any age. Fig 6A shows age-dependent decrease of the number of plaque-free Tg+YB-1 animals. Supposedly, the younger transgenic mice, the stronger effect of YB-1, which points to the highest efficiency of YB-1 at early stages of pathology development.

As a rule, double-staining of plaques with Thioflavin S and antibody to β-amyloid (1–42) demonstrated co-localization of these dyes, which points to the fact that plaques in 5XFAD mice are amyloid-like and β-amyloid-containing. Fig 6B shows a microphotograph with this double-staining described in detail.

### In OBX mice intranasally administered YB-1 penetrates into brain regions and enters neurons

**Fig 7:**
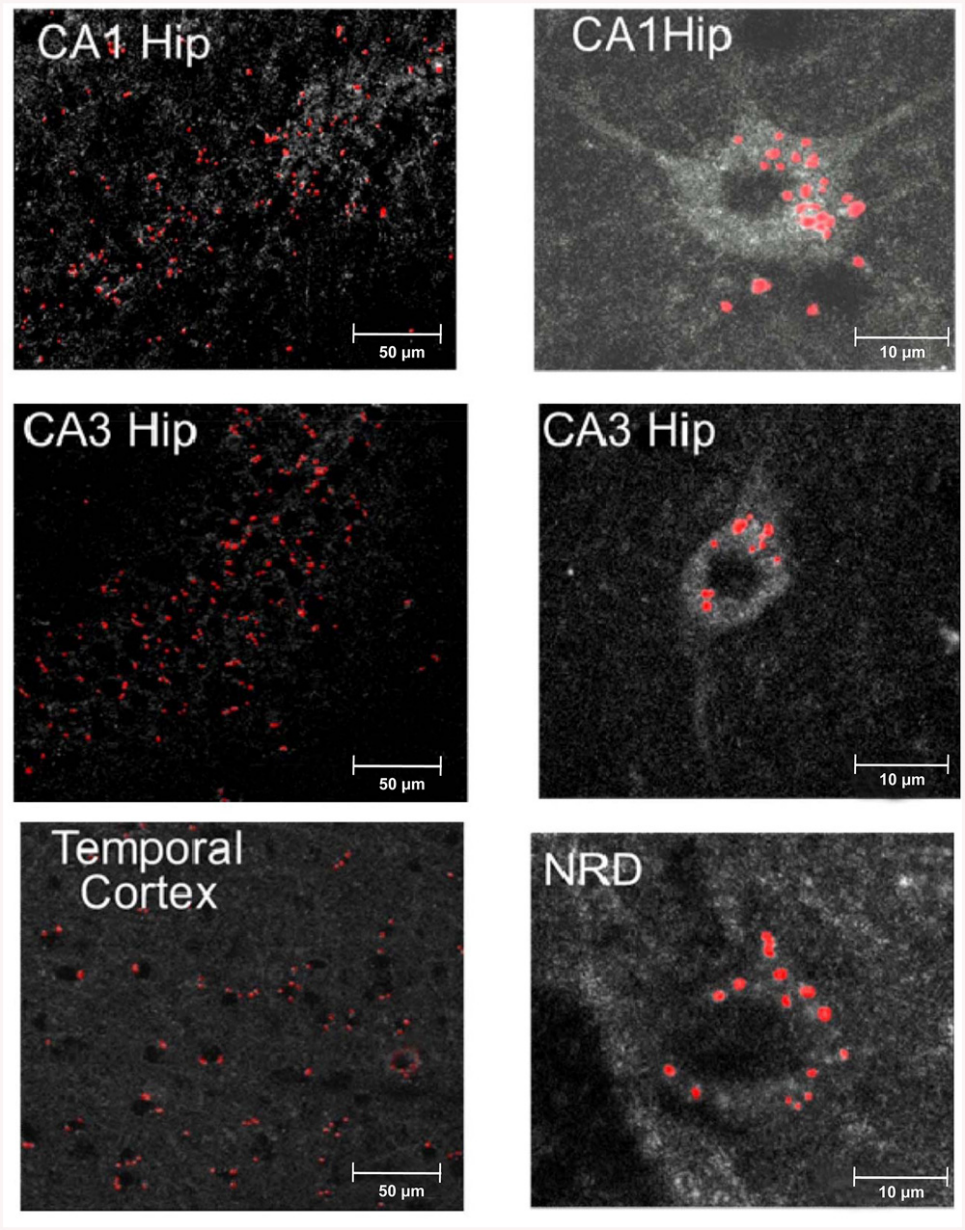
Penetration of intranasally-injected Cy3-labeled YB-1 into mouse brain regions. Left, microphotographs of the temporal cortex and the hippocampal areas CA1 and CA3. Red spots represent labeled YB-1. Right microphotographs are examples of possible YB-1 penetration into neurons of CA1, CA3, and NRD (the serotonin-synthesizing nucleus raphe dorsalis).

It was of special interest to address the issue of penetration of intranasally-administered YB-1 into the brain of OBX animals. The use of cyanine Cy3-conjugated YB-1 allowed observing (2 h after its intranasal administration) fluorescent granules in the brain, specifically, in the hippocampus, the temporal cortex, and the serotonin-synthesizing dorsal raphe nucleus of the brain stem, i.e., in AD-type neurodegeneration-affected regions. A part of the fluorescent label showed intracellular localization, as a rule, in the perinuclear space of neurons (Fig 7, right photos). Brain tissue samples from OBX mice treated with the identical dose of unlabeled YB-1 showed no fluorescence (data not shown).

### YB-1 penetrates into cells in primary neuronal culture of rat hippocampus

The above results on penetration of intranasally-administered YB-1 into neurons of some brain regions were verified using primary neuronal culture of rat hippocampus as a model system. These experiments used HA-YB-1 to rule out influence of hydrophobic Cy3 on penetration of the protein molecule into the cell. YB-1 penetration into cultured hippocampus cells was detected using primary anti-HA antibodies (Sigma, 1:1000) followed by secondary fluorescent antibodies conjugated with Alexa Fluor 488 (Invitrogen, 1:1000). After DAPI-staining of the nuclei, the samples were analyzed using a Leica TCS SPE confocal microscope.

**Fig 8:**
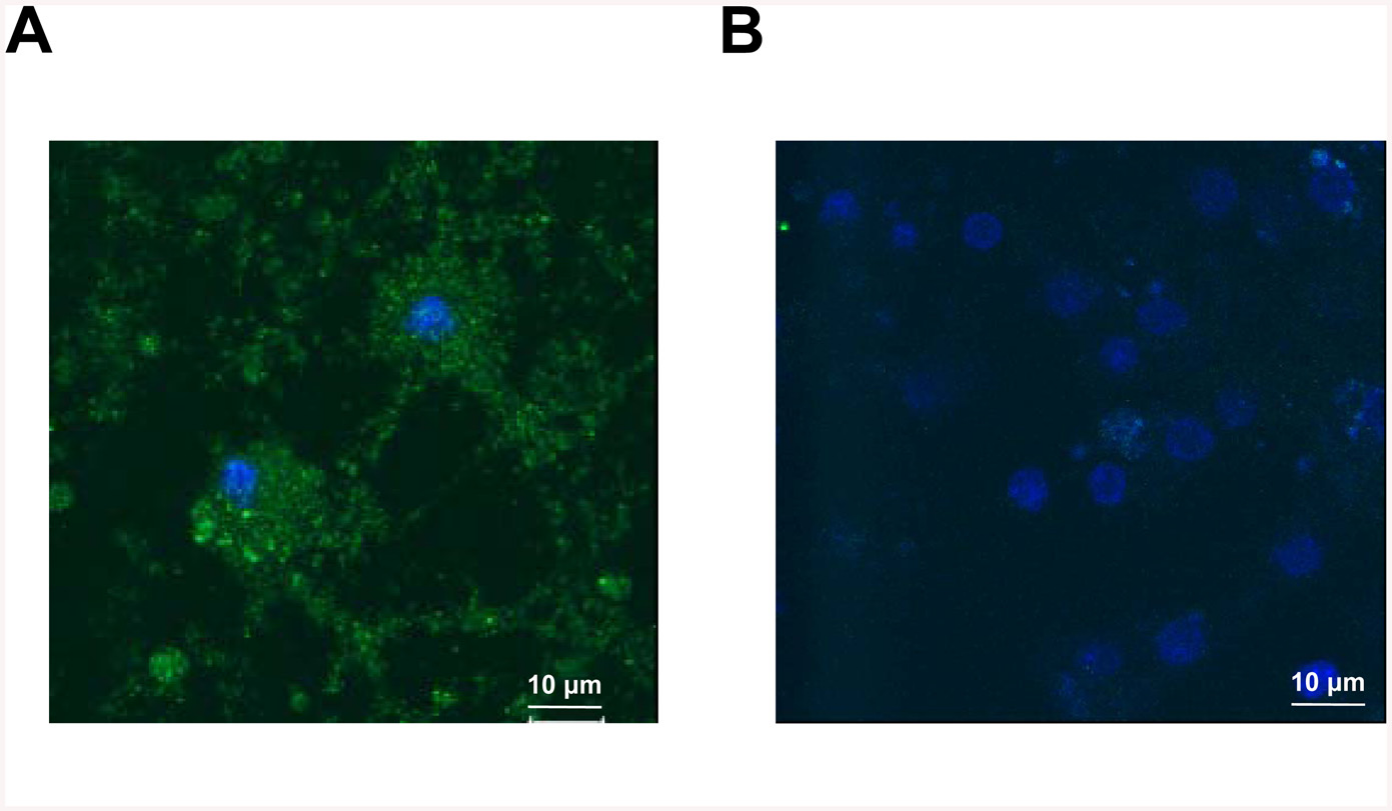
Distribution of HA-tagged YB-1 after its incubation with primary neuronal culture of rat hippocampus. The culture cells were incubated with HA-tagged YB-1 (**A**) or without it (**B**) for 2 h and stained with primary anti-HA antibodies (Sigma), followed by secondary antibodies conjugated with Alexa Fluor 488 (Invitrogen). Then samples supplemented with Pro Long Gold antifade reagent with DAPI were analyzed using a Leica TCS SPE confocal microscope. YB-1 gave green fluorescence, DAPI-stained nuclei gave blue fluorescence.

As shown by confocal microscopy, HA-YB-1 entered the neuronal culture cells (Fig 8A). It should be noted that distribution of Cy3-labeled YB-1 as big granules in neurons (Fig 7, right photos) differs from a more even distribution of HA-tag-labeled YB-1 in cells of primary neuronal culture of rat hippocampus (Fig 8A). This may result from an enhanced ability of YB-1 to aggregate when being conjugated with a hydrophobic dye. Indeed, *in vitro* experiments showed that Cy3-YB-1, unlike YB-1 alone, forms large aggregates in buffer solution (S3 Fig).

### Specificity of YB-1 penetration and its integrity in the cell

**Fig 9:**
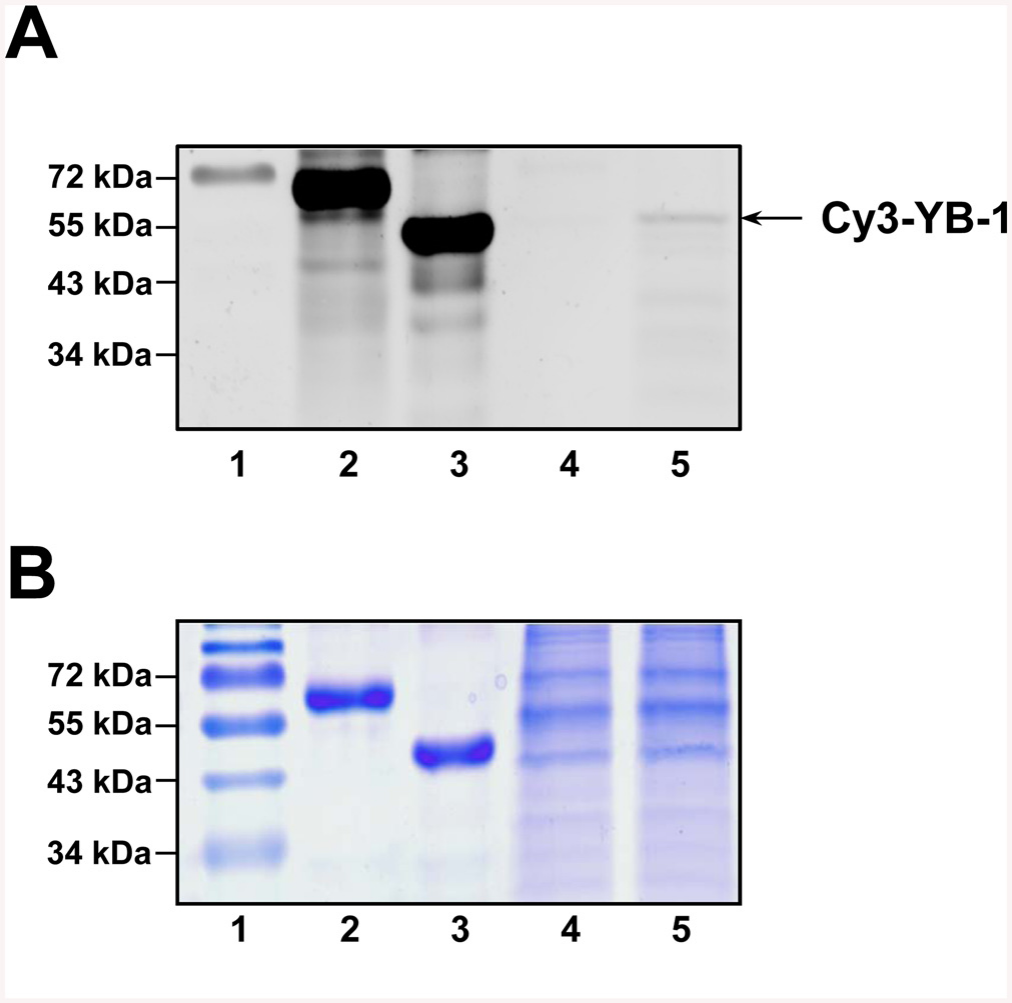
Penetration of Cy3-labeled YB-1 into primary neuronal culture of rat hippocampus. The culture cells were incubated with Cy3-labeled YB -1 or Cy3-labeled BSA for 2 h. Then cell lysate proteins were separated by SDS-PAGE and detected for fluorescence (A) and Coomassie brilliant blue staining (B). Lanes: 1, molecular mass markers with fluorophore-labeled 72 kDa marker (Thermo Scientific); 2, Cy3-labeled BSA; 3, Cy3-labeled YB-1; 4, Cell lysates of the neuronal culture after 2 h incubation with Cy3-labeled BSA; 5, Cell lysates of the neuronal culture after 2 h incubation with Cy3-labeled YB-1.

To address the specificity issue, the cultured cells were supplemented with either Cy3-labeled YB-1 or Cy3-labeled BSA used as a negative control and incubated for 2 h. After incubation, lysate proteins were separated by SDS-PAGE and detected for fluorescence (Fig 9A) and Coomassie staining (Fig 9B). A Typhoon FLA 9500 imaging system was used to detect fluorescent proteins (Fig 9A, lane 4 for Cy3-labeled BSA, lane 5 for Cy3-labeled YB-1). After incubation for 2 h, the Cy3-labeled YB-1 lysate still contained non-degraded YB-1, while that of Cy3-labeled BSA demonstrated the absence of specific fluorescence. This suggests that YB-1 not only specifically penetrates into neurons but also keeps its integrity there for at least two hours, while Cy3-labeled BSA is either incapable of penetrating into neuronal cells or rapidly degrades there.

### The YB-1 effect on in vitro β-amyloid fibril formation

**Fig 10:**
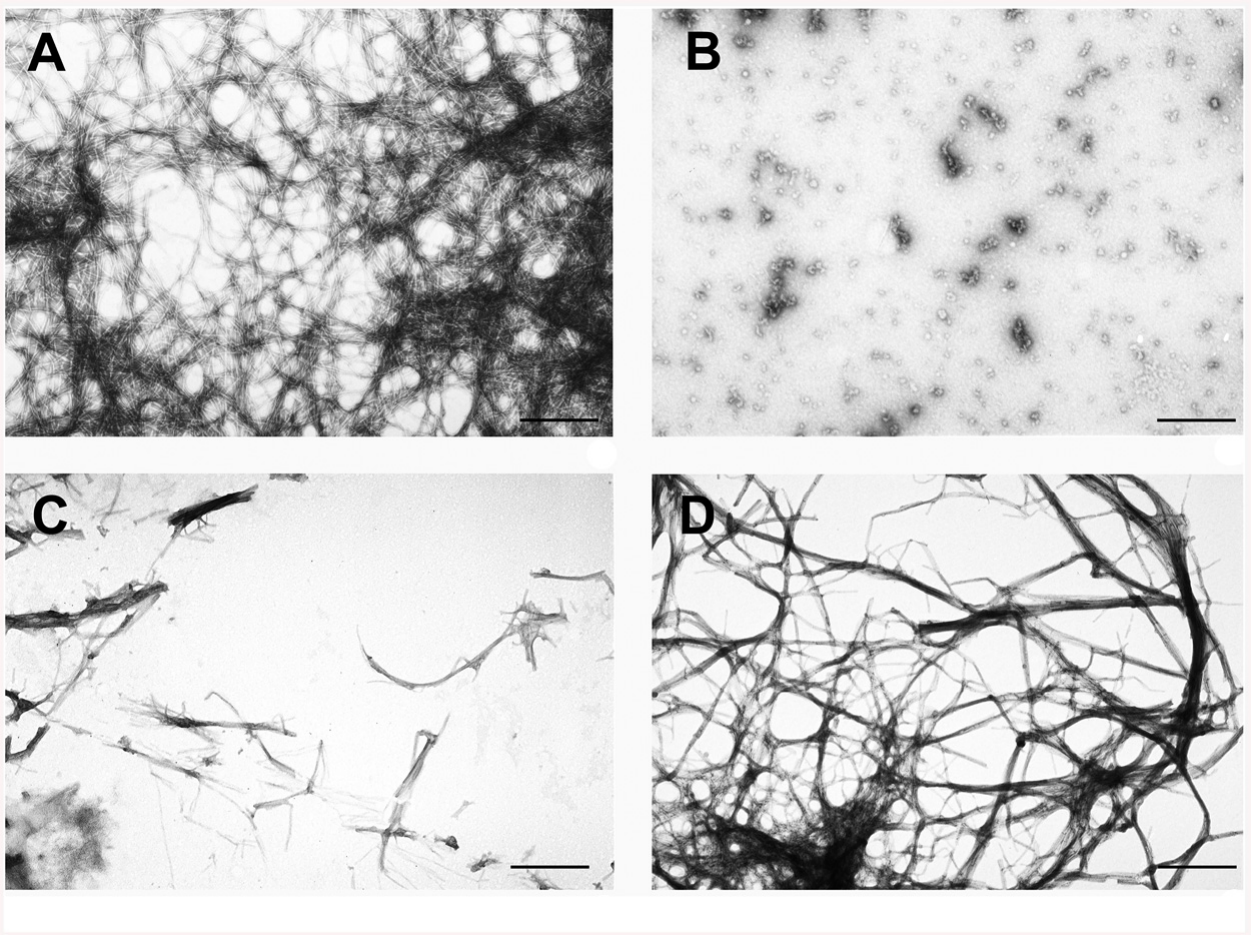
YB-1 prevents formation of *de novo* β-amyloid fibrils and contributes to disruption of already formed fibril nets. **A**, mature fibrils formed by the Aβ(1–42) peptide after 24 h incubation at 37°C in 10 mM imidazole, pH 7.0, 30 mM KCl; **B**, YB-1 after 24 h incubation in the same conditions; **C**, 24 h co-incubation of the Aβ(1–42) peptide and YB-1 in the same conditions; **D**, mature Aβ(1–42) fibrils were supplemented with YB-1 and incubated for 24 h in the same conditions.

Having shown that YB-1 decreases both the amount of β-amyloid in the brain of OBX and transgenic 5XFAD mice and density of their amyloid plaques, we verified by electron microscopy its effect on amyloid fibril formation by the Aβ(1–42) peptide in solution. As found, 24 h incubation of this peptide at 37°C in solution yielded linear fibrils (Fig 10A). In the YB-1 solution, typical amorphous aggregates were formed and remained unchanged even 24 h after incubation (Fig 10B). Co-incubation of the Aβ(1–42) peptide with YB-1 for 24 h gave dispersed short amyloid fibrils, instead of a dense fibril net (Fig 10C). We were also interested in establishing the effect of YB-1 on already formed Aβ(1–42) fibrils (i.e., after their 24 h incubation in conditions described in detail under Materials and Methods). It was shown that in the presence of YB-1 the pre-formed fibril net becomes less dense (Fig 10D).

This *in vitro* experiment supported our *in vivo* results showing the effect of YB-1 on the brain level of β-amyloid in OBX and transgenic 5XFAD mice and its influence on density of β-amyloid containing plaques.

### YB-1 interaction with β-amyloid in the brain

To learn whether there is direct or indirect interaction of YB-1 with β-amyloid, we studied brain slices from 6-months-old 5XFAD mice that had been daily intranasally injected with YB-1 for three weeks. The slices were stained with anti-β-amyloid antibody and anti-YB-1 antibody (for more details, see Materials and Methods).

**Fig 11:**
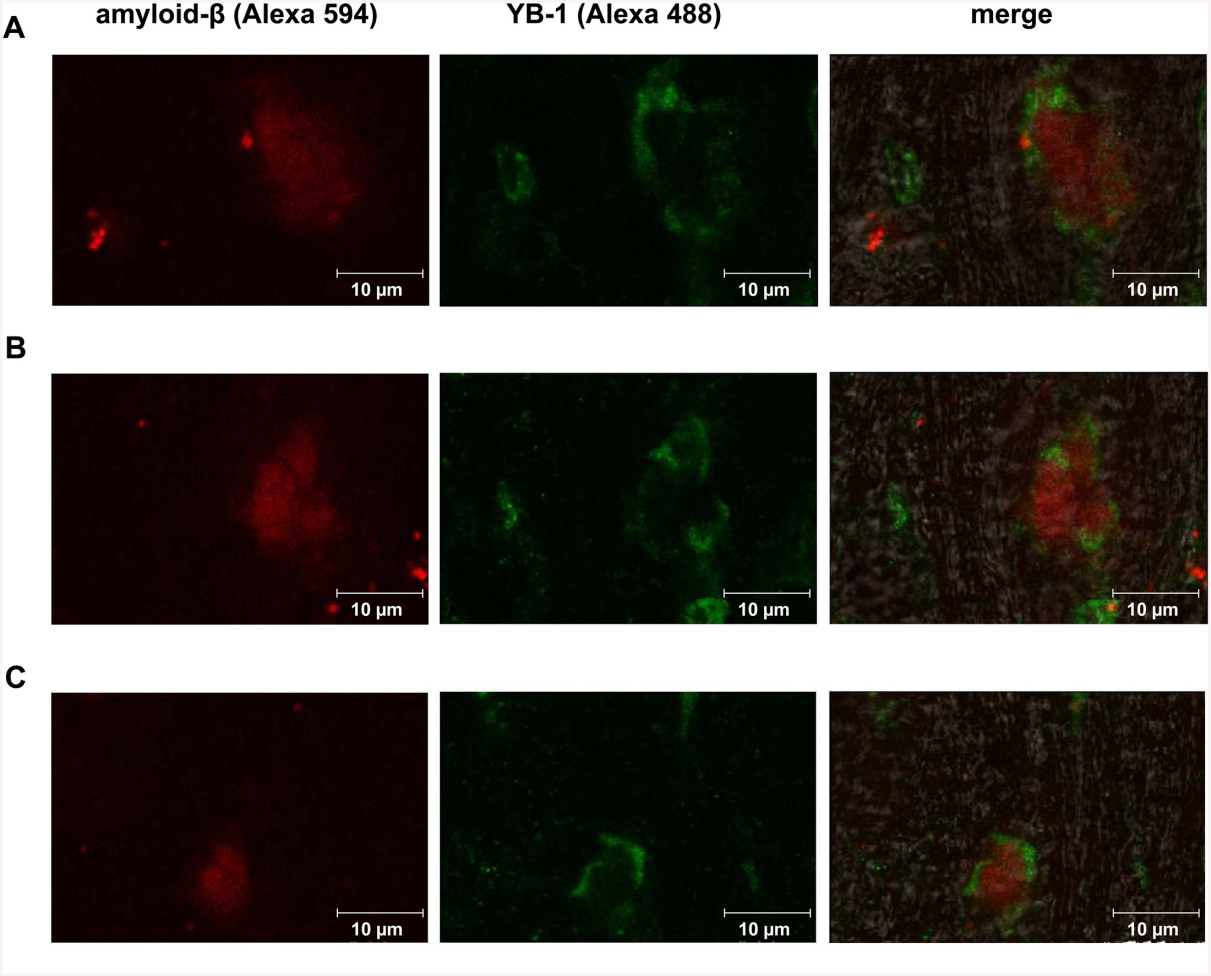
Distribution of YB-1 and β-amyloid in the brain of 5XFAD mice after subchronic intranasal administration of YB-1. **A, B, and C**, representative microphotographs of brain slices from transgenic 5XFAD mice after immunochemical double-staining with monoclonal Anti-Human β Amyloid Protein antibody (Sigma, 1:300) and secondary antibody with fluorophore Alexa 594 (Invitrogen, 1:300) (red fluorescent dye), as well as monoclonal anti-C-terminal YB-1 fragment antibody (Sigma, Y0396, 1:1000) and secondary goat anti-rabbit antibody labeled with Alexa 488 (Life Technology A-11008, 1:1000) (green fluorescence).

The histoimmunochemistry experiments proved close proximity, but not co-localization, of amyloid-β and YB-1 in brain slices (Fig 11A, 11B and 11C). It looks like YB-1 separates β-amyloid plaques from surrounding brain tissues.

## Discussion

We have demonstrated that in OBX mice full-length YB-1 and its fragment YB-1_1−219_ prevent impairment of spatial memory and protect neurons in the memory-related brain regions, particularly in the temporal cortex and the hippocampus. YB-1 treatment results in an increased density of neurons and a decreased number of cells with pathological changes (Table 1). Although the used YB-1 fragments had a certain protective effect on brain neurons in OBX mice, their effectiveness (especially that of the shortest fragment YB-1_52−129_) was not as high as that of the full-length protein. Importantly, unlike these fragments, full-length YB-1 did not form amyloid-like fibrils under physiological conditions [31], which probably explains its higher functional activity.

We used full-length YB-1 in transgenic animal experiments because of its higher effectiveness, as compared to its fragments. The effect of intranasal administration of this protein was studied using 6-months-old transgenic mice at a pronounced stage of AD with intensive plaque formation and memory loss. The positive effect of YB-1 was registered as better learning results testifying to improvement of short-term working memory. A similar improvement in patients with AD, if achieved, would have ameliorated both their everyday memory-related activities and communication habits.

Before addressing the issues of memory-protecting mechanisms of YB-1 in OBX mice and specifically, the YB-1 effect on the morphofunctional state of neurons in the temporal cortex and the hippocampus, we checked penetration of intranasally-administered YB-1 into these brain regions. The intranasally administered Cy3-labeled YB-1 was observed in both control and OBX animals. It was detected in the hippocampus, the temporal cortex, and the serotonin-synthesizing nucleus raphe dorsalis (NRD) of OBX mice, thereby indicating that YB-1 penetrates into the brain avoiding the hematoencephalic barrier (Fig 7, left photos).

The routes and mechanisms of YB-1 transport are not clear yet. There are several feasible mechanisms of its direct nose-to-brain delivery. In control animals, the protein may be carried to the brain, especially to its caudal regions, along the trigeminal nerve to bypass the hematoencephalic barrier. This may explain the presence of labeled YB-1 in NRD. Both in control and OBX mice, extracellular YB-1 may be carried by interstitial liquid. Possible mechanisms of its transport may involve the bulk flow and diffusion within perineuronal channels, perivascular spaces, or lymphatic channels directly connected with brain tissue or cerebrospinal fluid [32]. The intranasal drug administration attracts more and more attention as a therapeutically advantageous approach to neurodegeneration. Theoretically, drugs can enter the brain exclusively through the olfactory region that provides a possibility of extra- and intracellular drug delivery across the epithelial barrier directly to the arachnoid membranes, thus avoiding the blood flow. The precise mechanisms underlying intranasal protein delivery which may involve neuron terminals, interstitial fluid, or blood vessels, are subjects for future investigation.

Interestingly, in the brain, a part of labeled YB-1 was detected in the neuronal cytoplasm (Fig 7, right photos), thereby evidencing that labeled YB-1 is able to penetrate into neurons.

The YB-1 ability to penetrate through the cell membrane was investigated using a neuronal culture of rat hippocampus (Figs 8 and 9). We found that fluorescent dye-labeled as well as HA-tagged YB-1 penetrates into cells and localizes to granular structures in the cytoplasm. These results contribute to its functional importance since it reaches both intra- and extracellular targets. It is likely to use a yet unidentified cellular transport machinery, probably not only YB-1-specific, because the administered labeled protein Hsp70 showed a similar localization pattern in the brain regions [17].

The results reported here are similar to those of a previous study of Hsp70 [17]. The memory protecting activity of intranasally administered YB-1 and its effect on the morphofunctional state of neurons appeared to be similar to those of another stress protein, Hsp70. Similarity of their behavior implies existence of mutual mechanisms mediating realization of these effects. As known, both YB-1 and Hsp70 are stress proteins responsible for normal functioning of other proteins: Hsp70 acts as a chaperone, and YB-1 protects nucleus machinery under stress conditions [33]. This study first reports that YB-1 additionally performs the function of prevention of β-amyloid fibril formation. Interestingly, in the cell, the both proteins are associated with drug resistance [19,34], thereby contributing to cell viability in the presence of toxic agents, such as β-amyloid or hyperphosphorylated Tau-protein. Also, the both proteins are anti-apoptotic, and therefore they most likely assist neuronal viability under apoptosis-promoting AD conditions [19,35]. Lastly, their mutual target may be the Tau-protein, the state of which controls stability of microtubules, and hence, the axonal transport system in neurons. There is reason to believe that, like the Tau-protein, YB-1 and Hsp70 stabilize microtubules and are capable of performing Tau functions in the case it its inactivation [36,37]. As we have shown, both YB-1 and Hsp70 are brain-penetrant agents, and therefore, they may be useful for treatment of AD and tauopathies.

The major protein component of AD-typical plaques is the Aβ(1–42) peptide with a molecular mass of 4.2 kDa and a sequence of 42 amino acid residues that is a fragment of the large transmembrane amyloid precursor protein (APP). Supporters of the “amyloid hypothesis” consider it as an AD trigger that eventually leads to apoptosis induction and neuron death. Here, we demonstrate the effect of YB-1 and its fragments on the amount of β-amyloid in the brain of OBX mice. Since β-amyloid (1–40) is the major form observed in non-transgenic mice, while the 1–42 form is available in trace amounts only, in this study we used an ELISA kit mouse Aβ40 (Invitrogen) for Aβ(1–40) detection in OBX mice and a human Aβ kits to determine the level of Aβ(1–40) and Aβ(1–42) in transgenic 5XFAD mice. As found, in extracts of the cortex and hippocampus of OBX mice injected with YB-1 or its fragments, the level of Aβ(1–40) was almost as low as in control animals (Fig 3A). Interestingly, YB-1 affected β-amyloid in transgenic animals where a decreased level of plaque density in the cortex and hippocampus (Fig 5A–5C), as well as a decreased level of Aβ(1–40) and Aβ(1–42) (Fig 3B and 3C), were revealed. The highest physiological effect of YB-1 was observed in young transgenic mice. A contribution of YB-1 to suppression of amyloid plaque formation was additionally confirmed by the fact that the majority of plaque-free transgenic Tg+YB-1 animals were young (Fig 6A). YB-1 injections to transgenic mice not only reduced the number of amyloid plaques in their brains, but also structurally changed the plaques, i.e., caused their fragmentation and decreased density (Fig 5D–5F right column).

Direct evidence for the YB-1 effect on formation of β-amyloid fibrils was obtained from our *in vitro* experiments showing that YB-1 both prevented formation of *de novo* fibrils and contributed to disruption of already formed amyloid nets (Fig 10). Importantly, the remaining bundles of fibrils carried protein aggregates. We believe that these were YB-1 aggregates bound to β-amyloid and conclude that YB-1 prevents formation of neurotoxic β-amyloid fibrils.

The anti-fibril effect of YB-1 *in vitro* (Fig 10) explains the reduced density of amyloid plagues in YB-1-treated Tg mice (Fig 5). The immunohistochemical data suggested plaque incapsulation with YB-1, resulting in plaque separation from the surrounding tissue (Fig 11). It may be inferred that YB-1 involved in plaque incapsulation interacts with plaque-forming β-amyloid fibrills and destroys their fibrillar structure, thus exposing them to proteases. Determination of exact YB-1 binding epitopes in the β-amyloid structure that are critical for its preservation is a subject of further investigations.

Thus, we suggest that YB-1 *in vivo* reduces the neurotoxic effect of β-amyloid fibrils and increases the life span of neurons by blocking the increasing level of β-amyloid in the brain, decreasing propensity of new plaque formation, and disrupting already formed amyloid plaques.

Prevention of *de novo* fibril formation and partial disruption of pre-formed amyloid structures occurring in the presence of YB-1 may result from some distinctive properties of this protein. It was shown that YB-1 itself can form amyloid-like fibrils in conditions of high salt concentrations (1–2 M LiCl or KCl). However, unlike the majority of other amyloids, those formed by YB-1 are reversible and easily dissolve with salt concentrations going down to the physiological level [31]. On the other hand, it was shown that amyloid-forming proteins readily undergo co-polymerization in the course of fibril formation [38], and hence, they probably interact with one another. Together, this fact and our electron microscopy data (Fig 10) suggest that YB-1 may be involved either in interaction with the Aβ(1–42) peptide, thereby preventing its polymerization, or co-polymerization yielding vulnerable disruption-prone fibrils. This suggestion is yet to be verified by future studies.

Thus, we report that the recombinant protein YB-1 has demonstrated its neuroprotective effect in suppression of Alzheimer’s type neurodegeneration in OBX mice representing a model of sporadic AD, as well as in transgenic 5XFAD mice used as a model of cerebral amyloidosis. Our study sheds light on mechanisms of the protective properties of YB-1 in neurons of animals with modeled AD pathology. Specifically, we describe the ability of YB-1 to reduce the amount of β-amyloid and its anti-fibril assembly effect, which probably partially explains its potent neuroprotective activity in preventing cell death. However, taking into account not only the ability of exogenous YB-1 to penetrate into cells (Figs 7, 8 and 9), but also its preserved integrity there for at least two hours (Fig 9), we believe that there are other YB-1-mediated mechanisms, that regulate cell survival in conditions of AD-type neurodegeneration, to be discovered yet. This study provides the first evidence for the potential therapeutic benefit of YB-1 for AD prevention and treatment.

## Materials and Methods

### Animals

Male 3-months-old NMRI mice or transgenic 5XFAD mice weighing 20–25 g were used in experiments. The animals were housed in groups of 5–7 per cage in a climate-controlled room (21–23°C) with a natural light/dark cycle and free access to water and standardized food. During a sterile operation for the removal of olfactory bulbs (olfactory bulbectomy) mice were anaesthetized with Nembutal (40 mg/kg, i.p.) and 0.5% Novocaine for local anesthesia of the scalp. Both olfactory bulbs were aspirated through a burr hole with the coordinates L0; AP-2; H4. Sham-operated (SO) mice underwent the same procedure, except olfactory bulb ablation.

The generation of 5XFAD mouse line (TG6799) was described previously [39]. 5XFAD mice were purchased from JAX, maintained on a mixed SJL/C57Bl6 background and co-express the Swedish (K670N/M671L), Florida (I716V) and London (V717I) mutations in human APP(695) gene, and M146L and L286V mutations in PS1, both transgenes driven by Thy1 promoter. Transgenic mice were genotyped by conventional PCR of DNA purified from ear biopsies. The presence of transgenic cassette was detected using the primers 5’-AGGACTGACCACTCGACCAG-3’ and 5’-CGGGGGTCTAGTTCTGCAT-3’, and visualizing the 377 bp fragment as described previously [3]. This study was carried out in strict accordance with the “Regulations for Studies with Experimental Animals” (Decree of the Russian Ministry of Health of August 12, 1997, No.755). The protocol was approved by the Commission on Biological Safety and Ethics of the Institute of Theoretical and Experimental Biophysics, Russian Academy of Sciences (Permit # 173/k). All surgery was performed under sodium pentobarbital anesthesia, and all efforts were made to minimize suffering.

### Preparation and administration of YB-1

Preparation of YB-1 and its fragments was performed as described previously [31]. Two weeks after bulbectomy mice were daily intranasally injected with 4 μl solution of 1.6 μg/μl full-length YB-1 (YB-1_1−324_) or one of its two fragments, YB-1_1−219_ or YB-1_52−129_ (S1 Fig). The treatment lasted for three weeks: two weeks before and one during the training period. BSA- (1.6 μg/μl) or saline-administered OBX and transgenic mice were used as controls.

### Training and memory tests

Development of a navigational reflex in the Morris water maze was performed as described [17] and used as a training model. In experiments, a circular pool 80 cm in diameter filled with water (23°C) to a depth of 30 cm was conventionally divided into four equal sectors one of which, the target sector, contained a hidden save platform at a depth of 0.5 cm. The platform was 5 cm in diameter and invisible to swimming animals in milk-whitened water.

Both OBX and transgenic 5XFAD mice were given four pre-training trials to determine their latent period of finding an exposed (visible) platform in order to verify that they did not have motor or visual impairments that could affect the results of memory tests. A 5-day training period included four training trials daily with recording the latent periods of finding the invisible platform. It was followed by spatial memory tests implying the absence of the save platform for 1 min. The memory was considered to be good if the mice stayed in the target sector reliably longer or visited it more frequently than any indifferent sector.

The next day after the memory probe the experimental mice were perfused intracardially with 0.1 M phosphate buffer (pH 7.4) under terminal anesthesia with Nembutal (60 mg/kg, i.p.). Brains were rapidly removed in the cold, checked for bulbectomy completeness, and divided into hemispheres. Samples with incomplete bulbectomy or damaged frontal cortex were not used in histological and biochemical experiments, and their data were excluded from processing of the training and memory test results. Brain cortex and hippocampus tissue samples from the left hemisphere were frozen at –80°C and stored for a subsequent ELISA analysis of the β-amyloid level. The right hemisphere was fixed in 4% phosphate-buffered formaldehyde for a histological analysis.

A similar training in the Morris water maze followed by memory tests after intranasal injection of full-length YB-1 were performed using 6-months-old transgenic 5XFAD (TG6799) mice.

### Morphology

To investigate morphological and functional states of neurons from the temporal cortex and hippocampus of OBX mice, 15 μm-thick sections were subjected to Nissl staining with cresyl violet acetate (Sigma) and inspected under a Nikon Eclipse E200 optical microscope. The form and size of neurons, as well as intensity of their staining, were estimated. Only neurons with clearly seen contours, nucleus, and nucleoli were taken into account. One thousand cells from each area of each animal were analyzed. A PDP-12 digitizer computer system (Germany) was used for a comparative analysis of cellular compositions of the temporal cortex and the hippocampal areas CA1 and CA3. Neuronal abnormalities such as cytolysis, pyknosis, and vacuolization were assessed (S2 Fig), and the frequency of their occurrences was estimated *versus* the quantity of healthy neurons. The pyknomorphic neurons with a shrunken outer membrane were counted as dead ones. Cytolysis manifests itself by a damaged outer membrane. Vacuolization is characterized by the presence of cavities filled with liquid. The neuron density was determined per mm^2^. Neurons were counted in 10 view fields (x20).

### Thioflavine S staining and counting of amyloid plaques

A separate set of experiments was aimed to reveal the effect of intranasal injection of YB-1 on temporal plaque dynamics in the cortex and hippocampal areas of transgenic 5XFAD mice in dependence on age of animals. According to their age: 3 (n = 12), 4 (n = 11), 5 (n = 18), 6 (n = 17), and 7 (n = 16) months), transgene carriers (Tg) were divided into 5 groups, each further subdivided into two subgroups. In one subgroup, for a period of three weeks transgenic mice were intranasally injected with 4 μl solution of 1.6 μg/μl full-length YB-1 (Tg+YB-1 (n = 36)), while in the other mice of the same age were injected with saline and served as controls (Tg+saline (n = 37)). Two additional groups: 3 (n = 5) and 5 (n = 5) months were injected with BSA (4 μl of 1.6 μg/μl solution) for negative control (Tg+BSA). 24 hours after the last YB-1 administration the mice were deeply anaesthetized and perfused intracardially with saline followed by 4% phosphate-buffered (0.01 M, pH 7.4) formaldehyde. Brains were removed and fixed in freshly prepared 4% phosphate-buffered (0.01 M, pH 7.4) formaldehyde for two days, followed by rinsing in phosphate buffer (pH 7.4) for 12 h and then in water for another 12 h. Next, they were placed in 5% sucrose solution for 3 h and washed in deionized water twice for 1 h. A freezing microtome was used to prepare 10 μm-thick slices that were mounted on slides and left for 40 min in a humidified atmosphere for smoothing out. Thereafter, the samples were degreased with a 1:1 mixture of ethanol and chloroform for 60 min and washed in ethanol solutions of decreasing concentration (100%, 96%, 80%, and 40%) for 10–12 s each, followed by rinsing in distilled water. Prepared samples were stained with 0.1% Thioflavin S for 10 min in the dark and rinsed in 80% ethanol. Excess dye was removed by triple washing with distilled water. Fully dried slices were embedded in Apathy's gum syrup and used for microscopy. A Nikon digital camera was used to obtain microphotographs.

### Double-histoimmunostaining of β-amyloid plaques

A double-staining protocol was used to compare the β Amyloid Protein (17–24) immunoreactivity with Thioflavine-S staining of plaques. Monoclonal Anti-Human β Amyloid Protein (17–24) antibody (Sigma) recognizes residues 17 to 24 of β-amyloid, and was used at a final concentration of 10 μg/ml in TBS. The immunostaining procedure was performed on floating sections after Thioflavine-S staining as described above. Sections were washed in TBS, and then incubated for 30 min with 0.5% Triton X-100. Nonspecific binding was prevented by incubating the sections in Affini Pure Fab Fragments IgG (H+L) (Jackson ImmunoResearch Laboratories) in TBS for 2 h, before incubation with primary antibody at 4°C overnight. Sections were washed in TBS, and specific staining was revealed with a chicken anti-mouse antibody labeled with Alexa 594 (Invitrogen), sections were then mounted on Star Frost slides. Finally, the sections were coverslipped, and sealed with nail polish.

### β-amyloid detection by ELISA and immunohistochemistry

To detect the level of the β-amyloid peptide (1–40) by ELISA, some samples were treated as follows. Cortex and hippocampus tissues that had been stored at –80°C were weighed, thawed at room temperature, and homogenized in 2% CHAPS (3-[(3-Cholamidopropyl) dimethylammonio]-1-propanesulfonate in 20 mM Tris-HCl, pH 7.7) (4 ml per g of tissue) in the presence of protease inhibitors (10 μg/μl leupeptin, 10 μg/μl aprotinin, and 10 μg/μl AEBSF (4-(2-amino-ethyl)benzenesulfonyl fluoride hydrochlorid)). The homogenates were centrifuged at 21,000 *g* and 4°C for 30 min, cleared supernatants were stored at -80°C and thawed immediately before the ELISA analysis. The assay was conducted in accordance with ELISA kit manufacturer’s instructions (ELISA kit mouse Aβ40, Invitrogen). Optical density was measured at λ = 450 nm using an ELISA reader (Bio-rad).

For quantitative analysis of total levels of Aβ(1–40) and Aβ(1–42) in transgenic mice, the cortex and hippocampus from mice of the Tg+YB-1 (n = 6) and Tg+saline (n = 5) groups, as well as from Tg+BSA (n = 6) mice, were extracted in 8-fold volumes of cold 5 M guanidine HCl plus 50 mM Tris HCl (pH 8.0) buffer and centrifuged at 20,000 *g* for 1 h at 4°C to remove insoluble material. Final guanidine HCl concentrations were below 0.1 M. Supernatant fractions were analyzed using a human Aβ(1–40) ELISA kit (Invitrogen, KHB3481) and Aβ(1–42) ELISA kit (Invitrogen, KHB3441) according to the manufacturer’s protocol. Optical densities at 450 nm of each well were read using an ELISA reader (Bio-rad). Aβ40 or Aβ42 concentrations were determined by comparison with the respective standard curves. Aβ concentration value is expressed in nanograms per gram of brain tissue.

### Cy3 labeling of YB-1 and BSA

YB-1 and BSA labeling was performed as follows. 100 μl of YB-1 (5 mg/ml) or 100 μl of BSA (5 mg/ml) in 20 mM Hepes-KOH, pH 7.4, 0.5 M NaCl was incubated in the dark with 10 μl or 5.4 μl of Cy3 NHS ester solution (Biotech Industry Ltd., Russia) (6.6 mg/ml DMSO), respectively, at 22°C for 4 h, then dialyzed against 30 mM of potassium phosphate buffer, pH 7.4, containing 0.45 M NaCl. Concentrations of labeled proteins were estimated at 280 nm and 550 nm absorbance. The dye/protein molar ratio was approximately 1.2 in the final protein sample.

### Penetration of labeled YB-1 into brain regions

To assess the possibility of penetration of intranasally administered YB-1 into the brain, this protein was previously conjugated with the fluorescent dye cyanine Cy3 that displays the maximal absorption at 550 nm and the maximal irradiation at 570 nm. A 4 μg dose of labeled YB-1 was intranasally injected to SO (n = 4) and OBX (n = 5) mice that 2 h later were intracardially perfused. Their brains were rapidly removed in the cold, sectioned into 20 μm-thick slices, and inspected using a Leica TCS SP5 scanning confocal microscope. The samples were analyzed using 10x/0.3 NA or 20x/0.5 NA. HeNe laser was used to generate the 543 nm line for excitation, and pinholes were typically set to 1–1.5 airy units.

3–9 images were collected from non-overlapping fields at low power (total of 56 photomicrographs for analysis). Additionally, we collected 3 images of non-overlapping fields of labeled cells at high power (total of 36 photomicrographs for analysis). High-power images were collected from the temporal cortex, the hippocampal areas CA1 and CA3, and the nucleus raphe dorsalis (NRD).

### Preparation of primary neuronal culture

New-born (0-2-days-old) rats were treated with 70% ethanol and decapitated. The brain was rapidly placed into Petri dish with cold solution of HBSS (Gibco); using stereomicroscope, meningeal sheaths were thoroughly removed to isolate the hippocampus. Hippocampal tissue was supplemented with 500 μl of StemPro Accutase (Gibco) and incubated in a shaker for 15 min at 37°C. Using a Pasteur pipette, the sediment was re-suspended in the medium Neurobasal-A (Gibco) containing Penicillin-Streptomycin-Glutamine (1 ml per 99 ml medium), 50 mM Hepes (Gibco), 2% B-27 (Gibco). The cell suspension was twice centrifuged for 3 min at 1,000 rpm, the sediment was re-suspended in Neurobasal-A medium. 50 μl suspension aliquots were applied to PEI-treated (Sigma, 5 μg per 50 ml water) coverslips. Petri dishes with these coverslips were placed in a CO_2_-incubator (5% CO_2_, 37°C) for 2 h. After cell attachment, 2.5 ml Neurobasal-A medium was added, and the Petri dishes were returned to the CO_2_-incubator. Every 6–7 days, 1/3 of the medium was replaced by a fresh medium. The experiment was made 19 days after the beginning of cell cultivation in 5% CO_2_ at 37°C.

### Penetration of labeled YB-1 into primary neuronal culture of rat hippocampus

The 19-day culture of neurons was supplemented with the following Neurobasal-A medium-diluted agents: Cy3-YB-1 (4.75 μg per 1 ml medium) or Cy3-BSA (9.5 μg per 1 ml medium) or HA-YB-1 (5 μg per 1 ml medium), and incubated for 2 h in a CO_2_-incubator (5% CO_2_, 37°C). Then the cells on coverslips were rinsed in the solution for cell dissociation StemPro Accutase (Gibco) for 5 min.

The cells treated with HA-YB-1 were rinsed in PBS and immediately used for smear preparation. Briefly, cells were fixed in 3.7% paraformaldehyde-PBS for 20 min and permeabilized in PBS-0.5% Triton X-100 for 20 min. Coverslips were blocked with 5% goat serum in PBS for 30 min and incubated in 0.5% goat serum-PBS with primary anti-HA antibody (Sigma, 1:1000) for 1 h, followed by secondary antibody conjugated with Alexa Fluor 488 (Invitrogen, 1:1000). After addition of Pro Long Gold antifade reagent with DAPI, the samples were analyzed using a Leica TCS SPE confocal microscope.

Cells treated with Cy3-YB-1 or Cy3-BSA were lysed in buffer containing 10 mM Tris-HCl, pH 6.8, 1% SDS, 10% glycerol, 2% β-mercaptoethanol, 0.1% protease inhibitor cocktail. Proteins of cell lysates were resolved by 12% SDS-PAGE, and Cy3-labeled proteins were detected by a Typhoon FLA 9500 imaging system.

### Double-immunostaining of YB-1 and β-amyloid

A double-staining protocol was used to detect co-localization of YB-1 and β-amyloid immunoreactivity. The immunostaining procedure was performed on floating brain sections of 6- months-age transgenic 5XFAD mice after intranasal injections of YB-1 for three weeks. After β-amyloid immunostaining as described above, we used primary rabbit polyclonal antibody (Sigma, Y0396, 1:1000) against the C-terminal fragment of YB-1. The fixed slices were rinsed in PBS 3 times for 5 min. Nonspecific binding was prevented by incubating the sections for 2 h in 5% BSA. Sections were rinsed in PBS 3 times for 5 min and incubated with primary antibody at 4°C overnight, with subsequent triple rinsing in PBS. Specific staining was revealed using goat anti-rabbit antibody labeled with Alexa 488 (Life Technology A-11008, 1:1000) for 2 h, followed by triple rinsing for 5 min each. Further staining used antibody against β-amyloid and secondary antibody with fluorophore Alexa 594 (Invitrogen), as described under “Double-histoimmunostaining of β-amyloid plaques”. Sections were then mounted on Star Frost slides. Finally, the sections were coverslipped and sealed with nail polish. A Leica TCS SPE confocal microscope was used to inspect the samples.

### Formation of amyloid fibrils by the Aβ(1–42) peptide in vitro

Amyloid fibrils were formed by the Aβ(1–42) peptide (5 mg/ml) (Sigma-Aldrich) in a solution containing 30 mM KCl and 10 mM imidazole, pH 7.0, during 24 h at 37°C. Their amyloid properties were confirmed by binding to Thioflavin T and assessed using a Cary Eclipse (Varian) spectrofluorimeter (λ_ex_ 450 nm, λ_em_ 488 nm). YB-1 was added to the Aβ(1–42) peptide (w/w 1:1) before or after formation of the fibrils. The period of YB-1 incubation either with the Aβ(1–42) peptide or with its formed fibrils was 24 h at 37°C.

### Electron microscopy

A drop of either 0.1 mg/ml Aβ(1–42) peptide or YB-1 of the same weight concentration was applied to a carbon-coated colloidal film on a copper grid and negatively stained with 2% aqueous uranyl acetate. In experiments on amyloid formation by the Aβ(1–42) peptide in the presence of YB-1, concentration of each was 0.1 mg/ml. Samples were analyzed using a JEM-100B electron microscope (JEOL Ltd.).

### Statistical analysis

Data are given as the mean ±SEM. Significant differences between mean scores during training trials in the Morris water maze were assessed by two-way repeated measures ANOVA with Tukey’s post hoc analysis for multiple comparisons using group and trial day block number during training as sources of variation. Statistical analysis of the results of probe tests was carried out with ANOVA using group and sector of the maze as sources of variation. The preference for the target sector in comparison with each indifferent sector was assessed by post hoc analysis using a multiple range LSD test. ELISA-detected differences between experimental groups and age-dependent densities of amyloid plaques in transgenic 5XFAD mice, as well as changes in the morphofunctional state of neurons and neuronal density in OBX mice, were evaluated using the two-tailed Student’s t test.

## Supporting Information

S1 Fig The protein YB-1 (YB-1_1−324_) and its fragments. **A**, The domain structure of YB-1. A/P, the N-terminal Ala/Pro-rich domain; CSD, the cold shock domain; CTD, the C-terminal domain. **B**, Electrophoretic analysis of YB-1 and its fragments.
(PPTX)

S2 Fig Examples of neuronal pathologies in the brain of OBX mice. (PPTX)

S3 Fig YB-1 and Cy3-YB-1 aggregation characteristics. (PPTX)

S1 Table Values of the sector distinguishing factor for different groups of mice in memory. tests in the Morris water maze. (DOCX)

## References

[1] Bertoni-Freddari C, Fattoretti P, Casoli T, Caselli U, Meier-Ruge W (1996) Deterioration threshold of synaptic morphology in aging and senile dementia of Alzheimer's type. Anal Quant Cytol Histol 18: 209–213. 8790834

[2] Su JH, Anderson AJ, Cummings BJ, Cotman CW (1994) Immunohistochemical evidence for apoptosis in Alzheimer's disease. Neuroreport 5: 2529–2533. 7696596 10.1097/00001756-199412000-00031

[3] Peters OM, Shelkovnikova T, Tarasova T, Springe S, Kukharsky MS, Smith GA et al (2013) Chronic administration of Dimebon does not ameliorate amyloid-beta pathology in 5xFAD transgenic mice. J Alzheimers Dis 36: 589–596. 10.3233/JAD-130071 23645096

[4] Aleksandrova IY, Kuvichkin VV, Kashparov IA, Medvinskaya NI, Nesterova IV, Lunin SM et al (2004) Increased level of beta-amyloid in the brain of bulbectomized mice. Biochemistry (Mosc) 69: 176–180. 15000684 10.1023/b:biry.0000018948.04559.ab

[5] Bobkova N, Vorobyov V, Medvinskaya N, Aleksandrova I, Nesterova I (2008) Interhemispheric EEG differences in olfactory bulbectomized rats with different cognitive abilities and brain beta-amyloid levels. Brain Res 1232: 185–194. 10.1016/j.brainres.2008.07.036 18687318

[6] Bobkova NV, Nesterova IV., Medvinskaya NI., Aleksandrova IY., Samokhin AN., Gershovich JG., et al (2005) Possible role of olfactory system in Alzheimer’s disease genesis; Hanin L. AF, editor: Medimond.

[7] Bobkova NV (2010) Model of sporadic Alzheimer’s disease in bulbectomized animals.; Ugrumov M.V., editor. Moscow: Nauka.

[8] Bobkova NV, Nesterova IV, Nesterov VV (2001) The state of cholinergic structures in forebrain of bulbectomized mice. Bull Exp Biol Med 131: 427–431. 11550044 10.1023/a:1017907511482

[9] Borre Y, Lemstra S, Westphal KG, Morgan ME, Olivier B, Oosting R.S et al (2012) Celecoxib delays cognitive decline in an animal model of neurodegeneration. Behav Brain Res 234: 285–291. 10.1016/j.bbr.2012.07.007 22796600

[10] Douma TN, Borre Y, Hendriksen H, Olivier B, Oosting RS (2011) Simvastatin improves learning and memory in control but not in olfactory bulbectomized rats. Psychopharmacology (Berl) 216: 537–544. 21384104 10.1007/s00213-011-2245-0 PMC3140942

[11] Ostrovskaya RU, Gruden MA, Bobkova NA, Sewell RD, Gudasheva TA, Samokhin AN et al (2007) The nootropic and neuroprotective proline-containing dipeptide noopept restores spatial memory and increases immunoreactivity to amyloid in an Alzheimer's disease model. J Psychopharmacol 21: 611–619. 17092975 10.1177/0269881106071335

[12] Yehuda S, Rabinovitz S. (2014) Olfactory bulbectomy as a putative model for Alzheimer ‘: The protective role of essential fatty acids. PharmaNutrition 2: 12–14.

[13] Song C, Leonard BE (2005) The olfactory bulbectomised rat as a model of depression. Neurosci Biobehav Rev 29: 627–647. 15925697 10.1016/j.neubiorev.2005.03.010

[14] Hu J, Wang X, Liu D, Wang Q, Zhu LQ (2012) Olfactory deficits induce neurofilament hyperphosphorylation. Neurosci Lett 506: 180–183. 10.1016/j.neulet.2011.10.076 22094386

[15] Nesterova IV, Bobkova NV, Medvinskaya NI, Samokhin AN, Aleksandrova IY (2008) Morphofunctional state of neurons in the temporal cortex and hippocampus in relation to the level of spatial memory in rats after ablation of the olfactory bulbs. Neurosci Behav Physiol 38: 349–353. 10.1007/s11055-008-0048-5 18401724

[16] Bobkova N, Guzhova I, Margulis B, Nesterova I, Medvinskaya N, Samokhin A et al (2013) Dynamics of endogenous Hsp70 synthesis in the brain of olfactory bulbectomized mice. Cell Stress Chaperones 18: 109–118. 10.1007/s12192-012-0359-x 22836235 PMC3508132

[17] Bobkova NV, Garbuz DG, Nesterova I, Medvinskaya N, Samokhin A, Alexandrova I et al (2014) Therapeutic effect of exogenous hsp70 in mouse models of Alzheimer's disease. J Alzheimers Dis 38: 425–435. 10.3233/JAD-130779 23985416

[18] Miwa A, Higuchi T, Kobayashi S (2006) Expression and polysome association of YB-1 in various tissues at different stages in the lifespan of mice. Biochim Biophys Acta 1760: 1675–1681. 17045744 10.1016/j.bbagen.2006.08.027

[19] Eliseeva IA, Kim ER, Guryanov SG, Ovchinnikov LP, Lyabin DN (2011) Y-box-binding protein 1 (YB-1) and its functions. Biochemistry (Mosc) 76: 1402–1433. 22339596 10.1134/S0006297911130049

[20] Lyabin DN, Eliseeva IA, Ovchinnikov LP (2014) YB-1 protein: functions and regulation. Wiley Interdiscip Rev RNA 5: 95–110. 10.1002/wrna.1200 24217978

[21] Lu ZH, Books JT, Ley TJ (2005) YB-1 is important for late-stage embryonic development, optimal cellular stress responses, and the prevention of premature senescence. Mol Cell Biol 25: 4625–4637. 15899865 10.1128/MCB.25.11.4625-4637.2005 PMC1140647

[22] Lu ZH, Books JT, Ley TJ (2006) Cold shock domain family members YB-1 and MSY4 share essential functions during murine embryogenesis. Mol Cell Biol 26: 8410–8417. 16954378 10.1128/MCB.01196-06 PMC1636768

[23] Uchiumi T, Fotovati A, Sasaguri T, Shibahara K, Shimada T, Fukuda T et al (2006) YB-1 is important for an early stage embryonic development: neural tube formation and cell proliferation. J Biol Chem 281: 40440–40449. 17082189 10.1074/jbc.M605948200

[24] Frye BC, Halfter S, Djudjaj S, Muehlenberg P, Weber S, Raffetseder U et al (2009) Y-box protein-1 is actively secreted through a non-classical pathway and acts as an extracellular mitogen. EMBO Rep 10: 783–789. 10.1038/embor.2009.81 19483673 PMC2690452

[25] Fotovati A, Abu-Ali S, Wang PS, Deleyrolle LP, Lee C, Triscott J et al (2011) YB-1 bridges neural stem cells and brain tumor-initiating cells via its roles in differentiation and cell growth. Cancer Res 71: 5569–5578. 10.1158/0008-5472.CAN-10-2805 21730024

[26] Hanssen L, Frye BC, Ostendorf T, Alidousty C, Djudjaj S, Boor P et al (2011) Y-box binding protein-1 mediates profibrotic effects of calcineurin inhibitors in the kidney. J Immunol 187: 298–308. 10.4049/jimmunol.1100382 21606250

[27] Moiseeva NI, Rybalkina EYu, Vaiman AV, Malyshkina MA, Kim EP, Eliseeva IA, et al (2013) Effects of Extracellular YB-1 Protein on Cultured Cells of Human Breast Cancer. Biochemistry (Moscow) Supplement Series A: Membrane and Cell Biology 7: 21–28.

[28] Rauen T, Raffetseder U, Frye BC, Djudjaj S, Muhlenberg PJ, Eitner F et al (2009) YB-1 acts as a ligand for Notch-3 receptors and modulates receptor activation. J Biol Chem 284: 26928–26940. 10.1074/jbc.M109.046599 19640841 PMC2785380

[29] Bernstein HG, Lindquist JA, Keilhoff G, Dobrowolny H, Brandt S, Steiner J et al (2014) Differential distribution of Y-box-binding protein 1 and cold shock domain protein A in developing and adult human brain. Brain Struct Funct. 10.1007/s00429-014-0786-9 24817634

[30] Ables JL, Breunig JJ, Eisch AJ, Rakic P (2011) Not(ch) just development: Notch signalling in the adult brain. Nat Rev Neurosci 12: 269–283. 10.1038/nrn3024 21505516 PMC3159580

[31] Guryanov SG, Selivanova OM, Nikulin AD, Enin GA, Melnik BS, Serdyuk IN et al (2012) Formation of amyloid-like fibrils by Y-box binding protein 1 (YB-1) is mediated by its cold shock domain and modulated by disordered terminal domains. PLoS One 7: e36969 10.1371/journal.pone.0036969 22590640 PMC3348147

[32] Dhuria SV, Hanson LR, Frey WH 2nd (2010) Intranasal delivery to the central nervous system: mechanisms and experimental considerations. J Pharm Sci 99: 1654–1673. 10.1002/jps.21924 19877171

[33] Kim ER, Selytina AA, Buldakov IA, Evdokimova VM, Ovchinnikov LP, Sorokin AV, (2013) The proteolytic YB-1 fragment interacts with DNA repair machinery and enhances survival during DNA-damaging stress. Cell cycle 12: 3791–3803. 10.4161/cc.26670 24107631 PMC3905071

[34] Lianos GD, Alexiou GA, Mangano A, Rausei S, Boni L, Dionigi G et al (2015) The role of heat shock proteins in cancer. Cancer Lett 360: 114–118. 10.1016/j.canlet.2015.02.026 25721081

[35] Wang X, Chen M, Zhou J, Zhang X (2014) HSP27, 70 and 90, anti-apoptotic proteins, in clinical cancer therapy (Review). Int J Oncol 45: 18–30. 10.3892/ijo.2014.2399 24789222

[36] Chernov KG, Mechulam A, Popova NV, Pastre D, Nadezhdina ES, Skabkina OV et al (2008) YB-1 promotes microtubule assembly in vitro through interaction with tubulin and microtubules. BMC Biochem 9: 23 10.1186/1471-2091-9-23 18793384 PMC2557009

[37] Liang P, MacRae TH (1997) Molecular chaperones and the cytoskeleton. J Cell Sci 110 (Pt 13): 1431–1440. 9224761 10.1242/jcs.110.13.1431

[38] Kato M, Han TW, Xie S, Shi K, Du X, Wu LC et al (2012) Cell-free formation of RNA granules: low complexity sequence domains form dynamic fibers within hydrogels. Cell 149: 753–767. 10.1016/j.cell.2012.04.017 22579281 PMC6347373

[39] Oakley H, Cole SL, Logan S, Maus E, Shao P, Craft J et al (2006) Intraneuronal beta-amyloid aggregates, neurodegeneration, and neuron loss in transgenic mice with five familial Alzheimer's disease mutations: potential factors in amyloid plaque formation. J Neurosci 26: 10129–10140. 17021169 10.1523/JNEUROSCI.1202-06.2006 PMC6674618

