# Supplementary material for: The Y-Box Binding Protein 1 Suppresses Alzheimer’s Disease Progression in Two Animal Models
Source: PLoS One. 2015 Sep 22;10(9):e0138867. doi: 10.1371/journal.pone.0138867 (PMC4578864; doi:10.1371/journal.pone.0138867)
Supplement: S1 Fig — A, The domain structure of YB-1. A/P, the N-terminal Ala/Pro-rich domain; CSD, the cold shock domain; CTD, the C-terminal domain. B, Electrophoretic analysis of YB-1 and its fragments. (PPTX) [file pone.0138867.s001.pptx]

## Slide 1
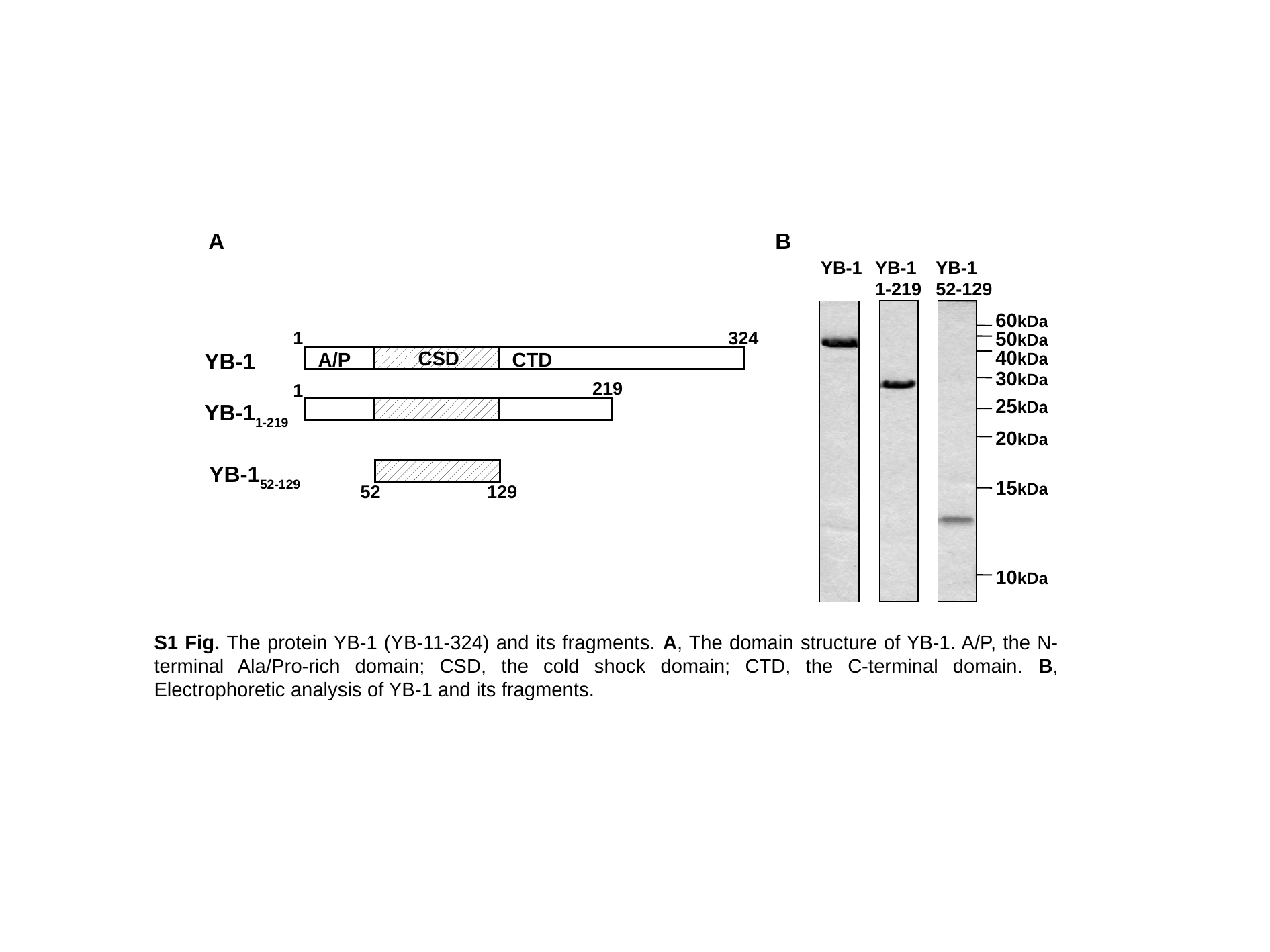

A
B
YB-1
1-219
YB-1
YB-1
52-129
60kDa
50kDa
40kDa
30kDa
25kDa
20kDa
15kDa
10kDa
324
1
YB-1
CSD
A/P
CSD
CTD
219
1
YB-11-219
 YB-152-129
52
129
S1 Fig. The protein YB-1 (YB-11-324) and its fragments. A, The domain structure of YB-1. A/P, the N-terminal Ala/Pro-rich domain; CSD, the cold shock domain; CTD, the C-terminal domain. B, Electrophoretic analysis of YB-1 and its fragments.
