# Supplementary figures and images for: The Y-Box Binding Protein 1 Suppresses Alzheimer’s Disease Progression in Two Animal Models
Source: PLoS One. 2015 Sep 22;10(9):e0138867. doi: 10.1371/journal.pone.0138867 (PMC4578864; doi:10.1371/journal.pone.0138867)

## Slide 1
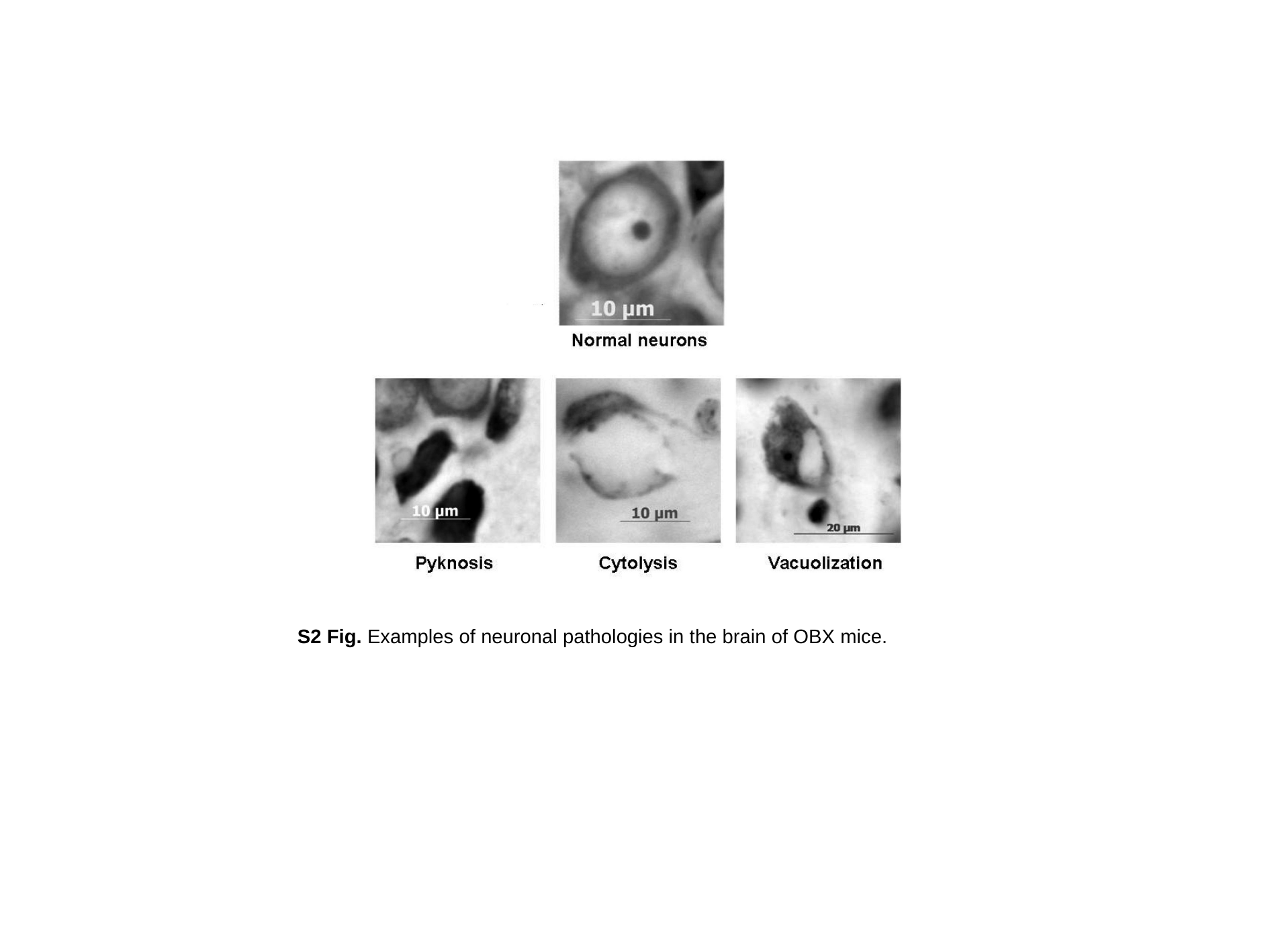

S2 Fig. Examples of neuronal pathologies in the brain of OBX mice.

Supplement: S2 Fig — (PPTX) [file pone.0138867.s002.pptx]
