# Supplementary material for: The Y-Box Binding Protein 1 Suppresses Alzheimer’s Disease Progression in Two Animal Models
Source: PLoS One. 2015 Sep 22;10(9):e0138867. doi: 10.1371/journal.pone.0138867 (PMC4578864; doi:10.1371/journal.pone.0138867)
Supplement: S3 Fig — (PPTX) [file pone.0138867.s003.pptx]

## Slide 1
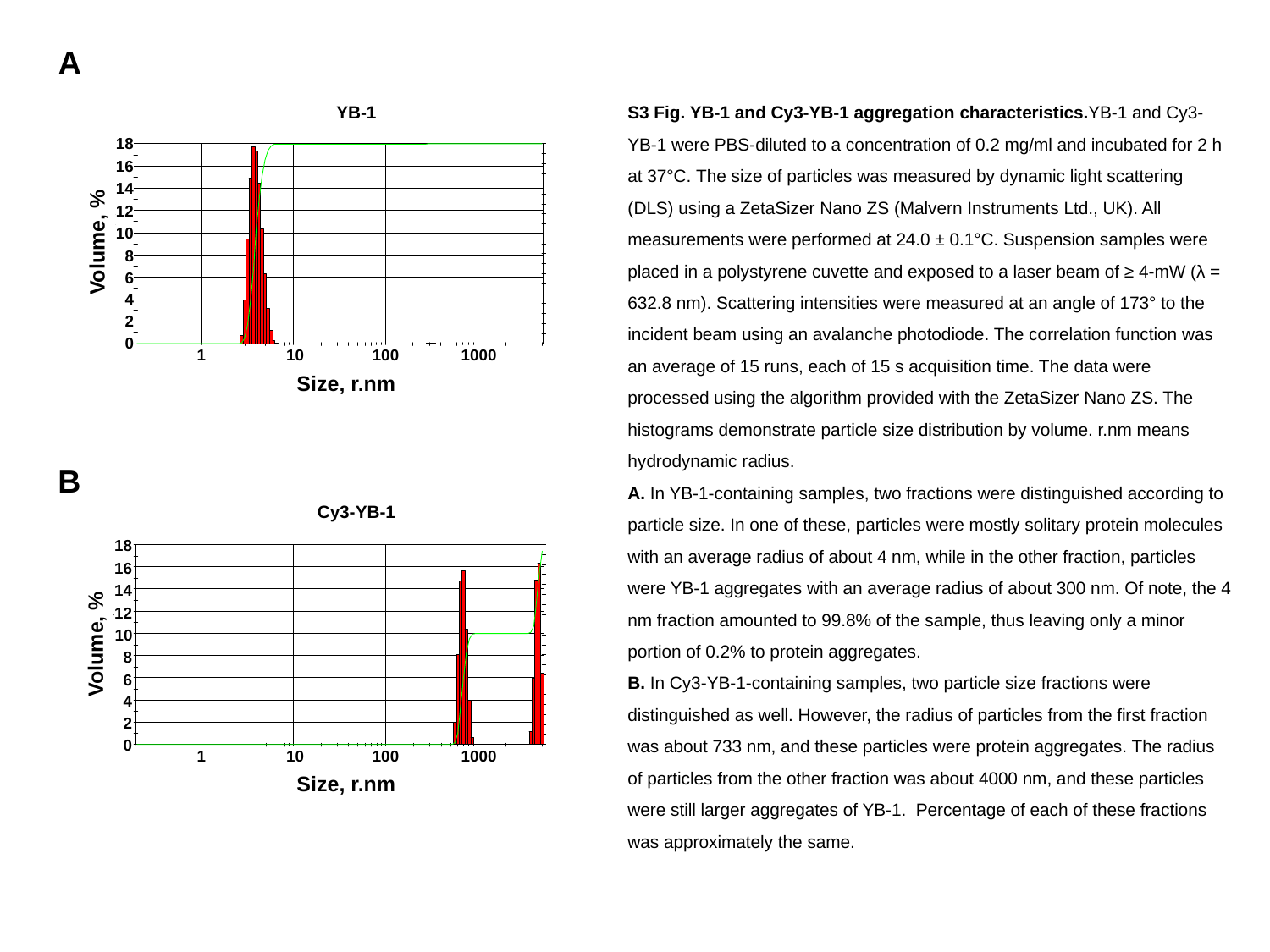

A
S3 Fig. YB-1 and Cy3-YB-1 aggregation characteristics.YB-1 and Cy3-YB-1 were PBS-diluted to a concentration of 0.2 mg/ml and incubated for 2 h at 37°С. The size of particles was measured by dynamic light scattering (DLS) using a ZetaSizer Nano ZS (Malvern Instruments Ltd., UK). All measurements were performed at 24.0 ± 0.1°С. Suspension samples were placed in a polystyrene cuvette and exposed to a laser beam of ≥ 4-mW (λ = 632.8 nm). Scattering intensities were measured at an angle of 173° to the incident beam using an avalanche photodiode. The correlation function was an average of 15 runs, each of 15 s acquisition time. The data were processed using the algorithm provided with the ZetaSizer Nano ZS. The histograms demonstrate particle size distribution by volume. r.nm means hydrodynamic radius.
A. In YB-1-containing samples, two fractions were distinguished according to particle size. In one of these, particles were mostly solitary protein molecules with an average radius of about 4 nm, while in the other fraction, particles were YB-1 aggregates with an average radius of about 300 nm. Of note, the 4 nm fraction amounted to 99.8% of the sample, thus leaving only a minor portion of 0.2% to protein aggregates.
B. In Cy3-YB-1-containing samples, two particle size fractions were distinguished as well. However, the radius of particles from the first fraction was about 733 nm, and these particles were protein aggregates. The radius of particles from the other fraction was about 4000 nm, and these particles were still larger aggregates of YB-1. Percentage of each of these fractions was approximately the same.
YB-1
18
16
14
12
Volume, %
10
8
6
4
2
0
1
10
100
1000
Size, r.nm
B
Cy3-YB-1
18
16
14
12
Volume, %
10
8
6
4
2
0
1
10
100
1000
Size, r.nm
