# Supplementary material for: The Y-Box Binding Protein 1 Suppresses Alzheimer’s Disease Progression in Two Animal Models
Source: PLoS One. 2015 Sep 22;10(9):e0138867. doi: 10.1371/journal.pone.0138867 (PMC4578864; doi:10.1371/journal.pone.0138867)
Supplement: S1 Table — (DOCX) [file pone.0138867.s004.docx]

**S1 Table. Values of the sector distinguishing factor for different groups of mice in memory tests in the Morris water maze.**

|  | Staying in Morris maze sectors, time | | Visits to Morris maze sectors, frequency | |
| --- | --- | --- | --- | --- |
| *Group* | *F(3.32)* | *P value* | *F(3.32)* | *P value* |
| SO + saline (n=9) | 45.52025 | 1.19E-11 | 44.87332 | 1.43E-11 |
| OBX + saline (n=9) | 2.507861 | 0.076512 | 2.33613 | 0.092325 |
| OBX+YB-1_1-324_ (n=9) | 27.57916 | 5.31E-09 | 9.107045 | 0.000167 |
| OBX+YB-1_1-219_ (n=9) | 19.27148 | 2.54E-07 | 10.67199 | 5.11E-05 |
| OBX+YB-1_52-129_ (n=9) | 5.392287 | 0.004054 | 3.328356 | 0.031761 |
| *Group* | *F(3.16)* | *P value* | *F(3.16)* | *P value* |
| OBX+BSA (n=5) | 1,987516 | 0.092125 | 2.057110 | 0.118235 |
